# The QTL *GNP1* Encodes GA20ox1, Which Increases Grain Number and Yield by Increasing Cytokinin Activity in Rice Panicle Meristems

**DOI:** 10.1371/journal.pgen.1006386

**Published:** 2016-10-20

**Authors:** Yuan Wu, Yun Wang, Xue-Fei Mi, Jun-Xiang Shan, Xin-Min Li, Jian-Long Xu, Hong-Xuan Lin

**Affiliations:** 1 National Key Laboratory of Plant Molecular Genetics, CAS Centre for Excellence in Molecular Plant Sciences and Collaborative Innovation Center of Genetics & Development, Shanghai Institute of Plant Physiology & Ecology, Shanghai Institute for Biological Sciences, Chinese Academic of Sciences, Shanghai, China; 2 University of the Chinese Academy of Sciences, Beijing, China; 3 Institute of Crop Sciences/National Key Facility for Crop Gene Resources and Genetic Improvement, Chinese Academy of Agricultural Sciences, Beijing, China; 4 Key Laboratory of Northern Japonica Rice Genetics and Breeding, Ministry of Education, Rice Research Institute, Shenyang Agricultural University, Shenyang, China; 5 Agricultural Genomics Institute, Chinese Academy of Agricultural Sciences, Shenzhen, China; 6 Shenzhen Institute of Breeding and Innovation, Chinese Academy of Agricultural Sciences, Shenzhen, China; University of California Berkeley, UNITED STATES

## Abstract

Cytokinins and gibberellins (GAs) play antagonistic roles in regulating reproductive meristem activity. Cytokinins have positive effects on meristem activity and maintenance. During inflorescence meristem development, cytokinin biosynthesis is activated via a KNOX-mediated pathway. Increased cytokinin activity leads to higher grain number, whereas GAs negatively affect meristem activity. The GA biosynthesis genes *GA20oxs* are negatively regulated by KNOX proteins. KNOX proteins function as modulators, balancing cytokinin and GA activity in the meristem. However, little is known about the crosstalk among cytokinin and GA regulators together with KNOX proteins and how KNOX-mediated dynamic balancing of hormonal activity functions. Through map-based cloning of QTLs, we cloned a GA biosynthesis gene, *Grain Number per Panicle1 (GNP1)*, which encodes rice GA20ox1. The grain number and yield of NIL-*GNP1*^TQ^ were significantly higher than those of isogenic control (Lemont). Sequence variations in its promoter region increased the levels of *GNP1* transcripts, which were enriched in the apical regions of inflorescence meristems in NIL-*GNP1*^TQ^. We propose that cytokinin activity increased due to a KNOX-mediated transcriptional feedback loop resulting from the higher *GNP1* transcript levels, in turn leading to increased expression of the GA catabolism genes *GA2oxs* and reduced GA_1_ and GA_3_ accumulation. This rebalancing process increased cytokinin activity, thereby increasing grain number and grain yield in rice. These findings uncover important, novel roles of GAs in rice florescence meristem development and provide new insights into the crosstalk between cytokinin and GA underlying development process.

## Author Summary

Grain number per panicle, a valuable agronomic trait for rice yield improvement, is profoundly affected by reproductive meristem activity. This activity, in turn, is controlled by transcriptional and plant hormone regulators, especially KNOX proteins and cytokinins. However, little is known about the roles of GAs in these processes in rice and how the regulatory network functions due to the complexity of crosstalk between plant hormone regulators. In this study, we identify a novel GA biosynthesis gene in rice and demonstrate its role in improving grain number and grain yield. We also propose that the KNOX-mediated cytokinin-GA activity rebalancing mechanisms regulate inflorescence meristem development and maintenance processes, providing a possible tool for high-yield rice breeding.

## Introduction

Rice panicle architecture, a valuable composite agronomic trait that includes grain number per panicle (GNP), panicle length and so on, is strongly associated with rice grain yield. GNP is one of the most important agronomic characteristics of ideal plant architecture [1]. To improve rice grain yields to meet the needs of the rapidly growing population, numerous studies have focused on identifying and cloning genes/QTLs contributing to rice panicle architecture development. Many genes and pathways have recently been identified, including transcriptional and plant hormone regulators that contribute to the reproductive meristem activity maintenance processes.

Cytokinins play a fundamental role in regulating reproductive meristem activity by promoting cell division [2]. *Grain number 1a (Gn1a)*, a cytokinin metabolism-related gene, encodes a cytokinin oxidase/dehydrogenase (OsCKX2) that catalyzes the degradation of active cytokinins in reproductive meristems. Thus, a null allele of *Gn1a* leads to improved rice grain yield through increased active cytokinin levels and reproductive meristem activity [3]. Another gene, *LONELY GUY (LOG)* encodes a cytokinin nucleoside 5’-monophosphate phosphoribohydrolase. *LOG* transcripts are specifically enriched in the apical regions of vegetative and reproductive meristems. LOG functions in the activation of cytokinin, catalyzing the conversion of inactive cytokinins to biologically active forms. Reduced active cytokinin levels in the meristem due to malfunctioning of cytokinin activation is likely responsible for the defective meristem activity in the *log* mutant [4]. In additions, the zinc finger transcription factor DROUGHT AND SALT TOLERANCE (DST) directly induces the expression of *OsCKX2* in the inflorescence meristems. The mutant allele *DST*^*reg1*^ reduces *OsCKX2* expression, thus increasing cytokinin levels in the inflorescence meristem, and therefore, the number of panicle branches and grains [5, 6].

Gibberellins (GAs) are crucial for plant growth and developmental processes, such as seed germination [7], grain setting [8] and so on. However, unlike cytokinins, GAs are primarily associated with high yield rice breeding due to their roles in plant height promotion. Most mutants or RNAi transgenic lines of GA biosynthesis genes, including *CPS*, *KS*, *KAO [9]*, *KO* [10], *GA20oxs* [11–13] and *GA3oxs* [14], show dwarfism phenotypes, which results in improved lodging resistance, a valuable trait for rice breeding under high inputs [15]. At the same time, transgenic-activated expression of GA catabolism genes, *GA2oxs*, also leads to dwarfism [16, 17]. However, GA signals are also active in inflorescence meristems. *OsGA20ox2*, *OsGA3ox2*, *Gα* and *SLR1* are highly expressed in inflorescence meristems and leaf primordia [18]. In maize, the expression domains of *GA2ox1* and *KN1* (a maize *KNOX* gene) overlap, mainly at the base of the shoot apical meristem. The *KNOX* gene *KN1* directly induces *GA2ox1* expression in reproductive meristems [19]. In tobacco and *Arabidopsis*, *GA20ox* expression could be directly excluded from the corpus of the shoot apical meristem [20, 21]. These findings suggest that GAs are detrimental to meristem activity. Although the importance of GAs in meristem establishment and maintenance has been recognized, the GA biosynthesis and regulatory networks underlying this process are largely unknown, and it also remains to be determined whether certain GA biosynthesis and regulatory genes can be useful for increasing grain number and yield in rice.

KNOX proteins are a class of homeodomain transcription factors that function in meristem establishment and maintenance. *OSH1* (a rice *KNOX* gene) can directly activate the expression of other *KNOX* paralogs (*OSH15*, for example) and itself. The positive autoregulation of *KNOX* genes and activation by cytokinin are both essential for meristem maintenance [22]. In rice and *Arabidopsis*, KNOX proteins can activate cytokinin biosynthesis in the meristems through the induction of genes encoding adenosine phosphate isopentenyltransferase (IPT). IPTs are important enzymes that convert ATP, ADP and AMP to the iP riboside 5’-triphosphate (iPRTP), iP riboside 5’-diphosphate (iPRDP) and iP riboside 5’- moophosphate (iPRMP) forms [23, 24]. As KNOX proteins reduce GA activity, they play an indispensable role in maintaining shoot apical meristem activity, probably by balancing cytokinin and GA activity in the meristems, increasing cytokinin levels and reducing GA levels [25, 26].

Here, we report the identification and characterization of a QTL, *Grain Number per Panicle1 (GNP1)*, which encodes rice GA biosynthetic protein OsGA20ox1. We propose that the upregulation of *GNP1* in the inflorescence meristems may increase cytokinin activity via a KNOX-mediated feedback regulation loop and increase GA catabolism activity through inducing the expression of *GA2oxs*. This process would result in increased cytokinin activity, rebalancing cytokinin and GA activity and increasing grain number and grain yield. These results provide insights into the mechanism underlying KNOX-mediated cytokinin and GA crosstalk during rice inflorescence meristem development, and they suggest that *GNP1* is a suitable target gene for high yield rice breeding.

## Results

### Positional Cloning of *GNP1*

To identify QTLs, we constructed two sets of reciprocal introgression lines (ILs) derived from a *japonica* rice variety Lemont (LT) and an *indica* variety Teqing (TQ), TQ-ILs and LT-ILs. In these two ILs, multiple QTLs for Grain Number per Panicle (GNP) were identified in Beijing and Sanya, respectively (S1 Table). Among these, QTLs affecting GNP in the RM227–RM85 region on chromosome 3 were detected in both TQ- and LT-ILs, suggesting that this QTL is stable for the grain number trait in rice. This QTL was designated *Grain Number per Panicle1 (GNP1)*.

**Fig 1:**
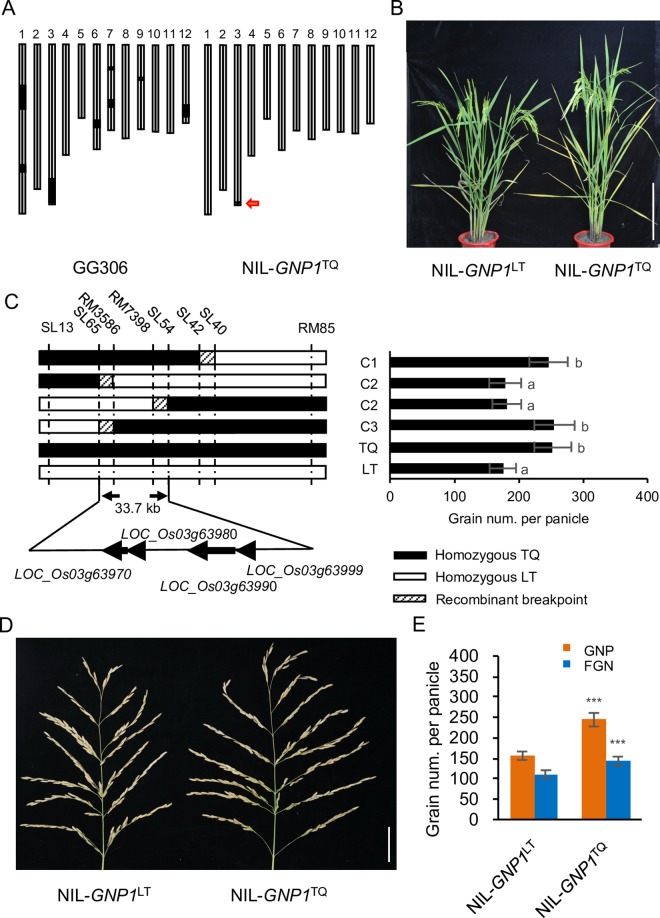
Characterization and map-based cloning of the *GNP1* QTL. (A) Graphical genotype of GG306 and NIL-*GNP1*^TQ^. NIL-*GNP1*^TQ^ contained the Teqing (TQ) allele at *GNP1* in the 66.1 kb region on chromosome 3. Black bars, genome fragments from TQ; white bars, genome fragments from LT. (B) Gross morphology of NIL-*GNP1*^LT^ and NIL-*GNP1*^TQ^. Scale bar, 40 cm. (C) High-resolution mapping of the *GNP1* locus. Black, white and lightly shaded rectangles indicate the homozygous TQ genotype, homozygous LT genotype and marker intervals containing recombination breakpoints, respectively. Different letters (a, b) indicate significant difference determined by the Fisher’s least significant difference (LSD) method at *p*-value < 0.01 (n = 40 plants). (D) Panicle morphology of NIL-*GNP1*^LT^ and NIL-*GNP1*^TQ^. Scale bar, 5 cm. (E) Comparison of GNP and FGN between NIL-*GNP1*^LT^ and NIL-*GNP1*^TQ^. Values are means ± s.d. (n = 18 plants). Asterisks represent significant difference determined by Student’s t-test at *p*-value < 0.001 (***), *p*-value < 0.01 (**), *p*-value < 0.05 (*).

From 201 LT-ILs, an IL named GG306 (BC_3_F_4_), containing chromosome segment RM227–RM85 from TQ and 92.6% of the genetic background of LT, was selected (Fig 1A) and backcrossed twice to LT. Self-pollination of BC_5_F_1_ plants heterozygous for this fragment resulted in heterozygous near-isogenic lines (NILs) with almost all of the genetic background of LT except for the introgressed segment (Fig 1A). The BC_5_F_2_ was successively self-pollinated several times to obtain segregating NIL-F_2_ (BC_5_F_3_, BC_5_F_4_ and BC_5_F_5_) populations for fine mapping of *GNP1* and construction of NILs, NIL-*GNP1*^LT^ and NIL-*GNP1*^TQ^ (Fig 1B).

An analysis of a BC_5_F_3_ population of 163 individuals derived by self-pollination of the BC_5_F_2_ heterozygotes at the region RM227–RM85 showed that the trait segregated as a single locus with a Mendelian ratio, which was confirmed by data from BC_5_F_4_ families (S1 Fig and S2 Table). Through map-based cloning of *GNP1*, we narrowed the *GNP1* locus down to a 33.7 kb region between SL65 and SL54 (Fig 1C and S2 Fig). This region contains four predicted genes (*LOC_Os03g63970*, *LOC_Os03g63980*, *LOC_Os03g63990* and *LOC_Os03g63999*, http://rice.plantbiology.msu.edu/cgi-bin/gbrowse).

### *GNP1* Increases Grain Number and Grain Yield

To further investigate the effects of the *GNP1* locus on grain number and other traits, we analyzed near-isogenic lines, NIL-*GNP1*^LT^ and NIL-*GNP1*^TQ^, in the LT genetic background, which only differed in the ~66.1 kb region containing *GNP1* derived from LT and TQ (Fig 1A). We observed a significant increase in the total grain number per panicle (GNP; +56%), filled grain number per panicle (FGN; +28%) and secondary branch number (SBN) in NIL-*GNP1*^TQ^ (Fig 1D, Fig 1E and S3F Fig, the same pattern in SBN between LT and TQ (S3G Fig)), but only a small increase in plant height (+8%; Fig 1B and S3A Fig), a slight decrease in grain length (-4%; S3B Fig), grain width (-5%; S3C Fig) and 1,000-grain weight (-12%; S3D Fig) and no effect on panicle length (S3E Fig) and primary branch number (S3F Fig the same pattern in PBN between LT and TQ (S3G Fig)) compared with the NIL-*GNP1*^LT^ isogenic control in plants grown in Shanghai. These results indicate that the *GNP1*^TQ^ locus in NIL-*GNP1*^TQ^ has pleiotropic effects on rice development, primarily on inflorescence development, especially secondary branch number and grain number.

To determine whether *GNP1*^TQ^ affects grain yield, we evaluated the grain yields of NIL-*GNP1*^TQ^ and the isogenic control (Lemont), together with other related traits. In different fields, the grain number was still substantially higher in NIL-*GNP1*^TQ^ than in the control, leading to a significant increase in grain yield (5.7–9.6%) despite the slightly reduced grain weight (Table 1 and S3 Table). These results suggest that the *GNP1*^TQ^ locus can potentially be used in high yield rice breeding.

**Table 1:** Performance of agronomic traits for NIL-*GNP1*^TQ^ and isogenic control (Lemont) across different environments.

Env.	Genotype	FGN	GNP	TGW	GL	GW	GY
BJ	Lemont	139.3	173.3	25.1	9.1	2.6	7.89
	NIL-*GNP1*^TQ^	189.0**	226.8*	23.4*	8.9*	2.5	8.43
	GYI						6.84%
NN	Lemont	154.4	190.2	25.2	9.2	2.7	8.49
	NIL-*GNP1*^TQ^	189.3*	252.6**	23.1*	9.0	2.6	8.97
	GYI						5.65%
JZ	Lemont	143.4	188.8	25.6	9.1	2.6	9.36
	NIL-*GNP1*^TQ^	161.7*	274.8***	23.4*	8.8*	2.5	9.90
	GYI						5.76%
PX	Lemont	101.3	119.7	26.3	9.4	2.6	9.69
	NIL-*GNP1*^TQ^	129.8*	184.5*	23.6**	9.2*	2.5	10.62
	GYI						9.60%
SY	Lemont	122.8	148.8	26.2	9	2.6	8.25
	NIL-*GNP1*^TQ^	171.7**	205.3**	24.3*	8.7*	2.5	8.97
	GYI						8.73%

FGN: filled grain number per panicle, GNP: grain number per panicle, TGW: thousand grain weight (g), GL: grain length (mm), GW: grain width (mm), GY: grain yield kg/13.3 m^2^, GYI: GY increase compared to Lemont, BJ: Beijing, NN: Nanning, JZ: Jingzhou, PX: Pingxiang, SY: Sanya.

*, ** and *** represent significance differences at *p*-value≤ 0.05, 0.01, and 0.001, respectively.

### *GNP1* Encodes Rice GA20-oxidase 1

According to the mapping results, *LOC_Os03g63980* and *LOC_Os03g63990* are predicted to encode transposon and retrotransposon proteins, *LOC_Os03g63999* encodes a small peptide with unknown function and *LOC_Os03g63970* encodes GA 20-oxidase 1, which is thought to catalyze the conversion of GA_12_ to GA_20_ within a multi-step process. Therefore, *LOC_Os03g63970* is the most likely candidate for the *GNP1* locus.

We sequenced the promoter (2 kb before ATG) and *LOC_Os03g63970* in both TQ and LT. The two parents exhibited base differences at 21 positions in the promoter region, including 17 single-base substitutions, as well as two single-base and two multi-base insertions and deletions. The coding region contains two single-base substitutions, one of which leads to an amino acid substitution (S4 Fig). These results suggest that the sequence differences in the promoter and coding region of this gene might lead to changes in gene expression levels and protein function and may help increase grain number in NIL-*GNP1*^TQ^.

**Fig 2:**
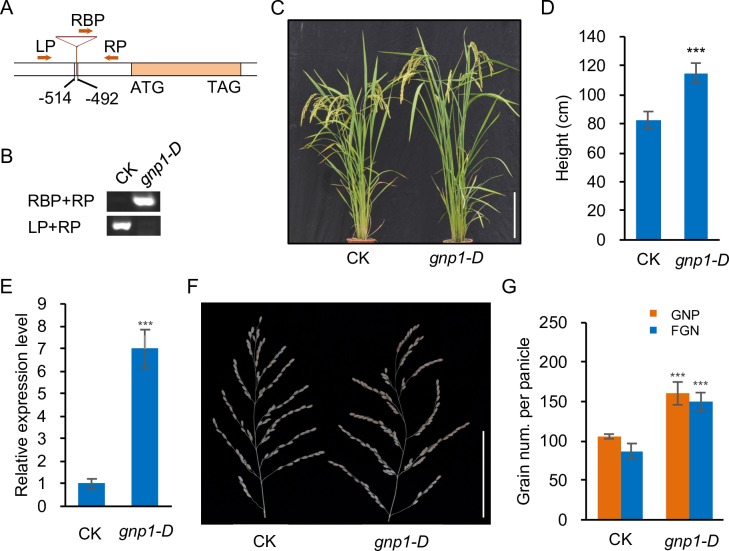
Characterization of the *GNP1* T-DNA gain-of-function mutant. (A) Position of T-DNA insertion in the *gnp1-D* mutant. The primers used for PCR screening of insertional mutants are marked with orange arrows. Orange box represents the coding region, inverted triangle indicates T-DNA insertion and negative numbers indicate the detailed insertion position obtained through DNA sequencing relative to the start codon ATG. (B) PCR screening of homozygous insertional *gnp1-D* mutant and the control (CK). (C) Gross morphology of *gnp1-D* and CK. Scale bar, 20 cm. (D) Comparison of plant height between *gnp1-D* and CK. Values are means ± s.d. (n = 10 plants). (E) Relative expression levels of *GNP1* in the flag leaves of *gnp1-D* and CK. Values are means ± s.d. (n = 4). (F) Panicle morphology of *gnp1-D* and CK. Scale bar, 10 cm. (G) Comparison of GNP and FGN per panicle between *gnp1-D* and CK. Values are means ± s.d. (n = 10). Asterisks represent significant difference determined by Student’s t-test at *p*-value < 0.001 (***).

To validate this hypothesis, we obtained the *LOC_Os03g63970* T-DNA gain-of-function mutant *gnp1-D* from the Rice T-DNA Insertion Sequence Database. TAIR-PCR screening showed that the T-DNA was inserted at position -514 to -492 of the *LOC_Os03g63970* promoter relative to the start codon ATG (Fig 2A), which constitutively induces the expression of *LOC_Os03g63970* throughout the plant. We analyzed traits of the homozygous *gnp1-D* mutant and control via PCR with specific primers designed based on the insertion sequence (Fig 2A and Fig 2B), finding a significant increase in plant height (Fig 2C and Fig 2D) with increasing *LOC_Os03g63970* expression in flag leaves (Fig 2E). Interestingly, a substantial increase in GNP (+51.5%) and FGN (+71.6%) were also observed (Fig 2F and Fig 2G). These results suggest that *LOC_Os03g63970* is the gene for *GNP1* and that the increased *GNP1* expression in this mutant might influence GA biosynthesis during rice panicle meristem development.

**Fig 3:**
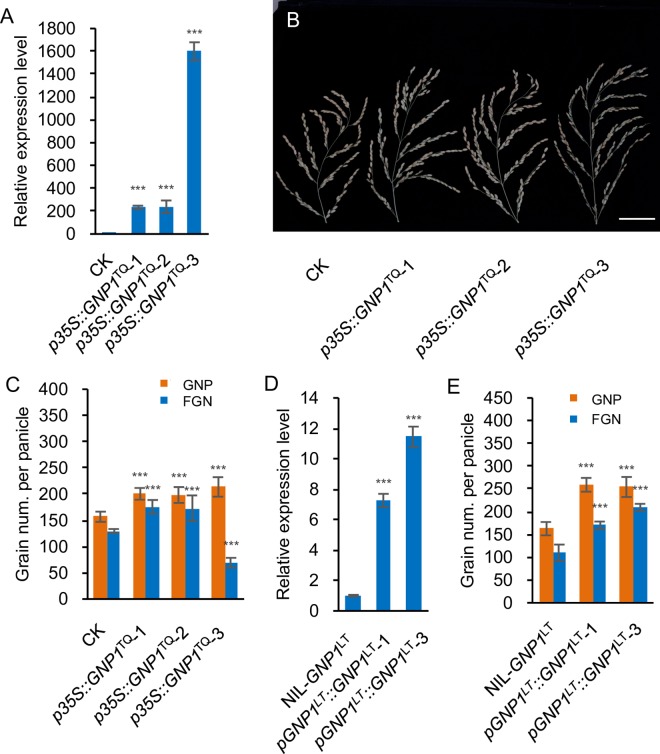
Effect of overexpression of *GNP1* in transgenic rice lines. (A) Relative expression levels of *GNP1* in the flag leaves of three independent *GNP1*^TQ^ overexpression lines and CK (transgenic negative control). Values are means ± s.d. (n = 4). (B) Panicle morphology of three independent *GNP1*^TQ^ overexpression lines and CK. Scale bar, 5 cm. (C) Comparison of GNP and FGN between three independent *GNP1*^TQ^ overexpression lines and CK. Values are means ± s.d. (n = 10). (D) Relative expression levels of *GNP1* in the flag leaves of two independent *pGNP1*^LT^::*GNP1*^LT^ overexpression lines and the recipient NIL-*GNP1*^LT^. Values are means ± s.d. (n = 4). (E) Comparison of GNP and FGN between two independent *pGNP1*^*LT*^::*GNP1*^LT^ overexpression lines and the recipient NIL-*GNP1*^LT^. Values are means ± s.d. (n = 10). Asterisks represent significant difference determined by Student’s t-test at *p*-value < 0.001 (***).

We then constructed a binary vector harboring the *GNP1*^TQ^ coding sequence (CDS) driven by a C*aMV 35S* promoter, which we used to transform *japonica* rice (*O*. *sativa* L.) variety Zhonghua 11 (ZH11), whose *GNP1* CDS matches that of LT. *GNP1* was expressed at levels several hundred- to over a thousand-fold that of CK (transgenic negative control) in flag leaves (Fig 3A). Compared with CK, the GNP of line *p35S*::*GNP1*^TQ^-3 increased by 36.3%, accompanied with hugely increased height (S5A and S5B Fig) and greatly increased sterility, while lines *p35S*::*GNP1*^TQ^-1 and *p35S*::*GNP1*^TQ^-2 had significantly increased GNP (FGN) by 27.8% (35.5%) and 26.5% (33.4%) (Fig 3B and Fig 3C), and slightly increased height (S5A and S5B Fig). These results indicate that the expression disturbances associated with the promoter activity variations at the *GNP1* locus are responsible for the phenotypic variation in GNP and plant height with a dose-dependent manner and a very high expression level of *GNP1* may have a negative effect on seed setting rate.

Then, in order to find out whether decreased expression of *GNP1* could show some negative effect on grain number phenotype, we transformed ZH11 with the mimic artificial microRNA oligo sequence designed for *GNP1* silencing driven by the C*aMV 35S* promoter. Interestingly, the grain number of six transgenic-positive independent lines increased (S6A Fig), which was negatively correlated with *GNP1* expression (S6B Fig). These lines also had reduced plant height (S6C and S6D Fig). These results indicate that the reduced expression of *GNP1* might contribute to attenuated GA biosynthesis activity, leading to reduced GA levels and partially reducing the negative effects of GAs on maintaining inflorescence meristem activity [26], which might be responsible for the higher grain number in these mimic artificial miRNA transgenic lines.

To further confirm the function of *GNP1*^LT^ CDS, we transformed NIL-*GNP1*^LT^ with *GNP1*^LT^ CDS driven by the *GNP1* promoter from Lemont (*pGNP1*^LT^). Similar to *gnp1-D* gain-of-function mutant and *GNP1*^TQ^ overexpression lines, as the expression level of *GNP1* increased (up to nearly ten-fold compared to the control; Fig 3D), we observed an increase in GNP and FGN (Fig 3E), as well as plant height (S5C Fig). These results indicate that both *GNP1*^LT^ and *GNP1*^TQ^ could affect panicle development.

**Fig 4:**
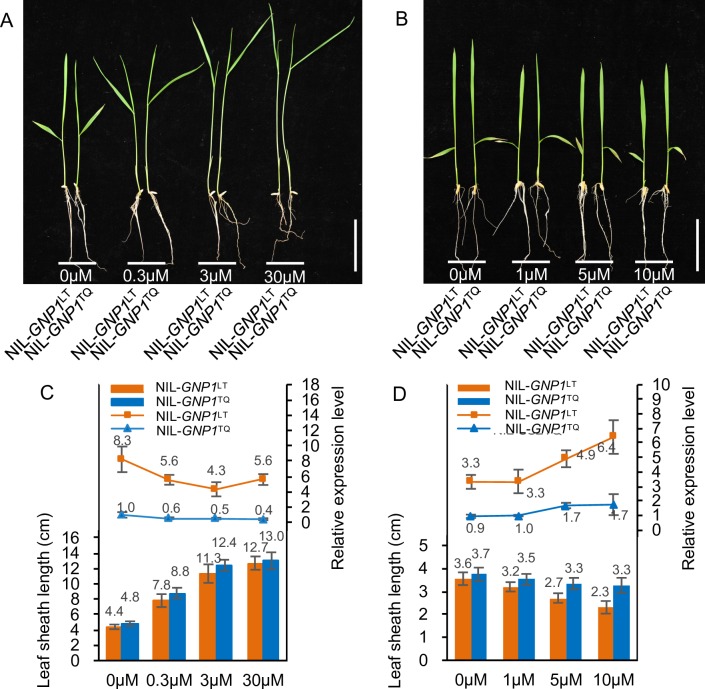
The response of *GNP1* to various treatments. (A) Dose-dependent response of leaf sheath length in NIL-*GNP1*^LT^ and NIL-*GNP1*^TQ^ to GA_3_ treatment. Scale bar, 5 cm. (B) Dose-dependent response of leaf sheath length in NIL-*GNP1*^LT^ and NIL-*GNP1*^TQ^ to uniconazole-P treatment. Scale bar, 5 cm. (C) Comparison of leaf sheath length (n = 48) together with the relative expression levels of *GNP1* in treated seedling leaf sheaths (n = 6, each with 8 plants) among different GA_3_ treatments for NIL-*GNP1*^LT^ and NIL-*GNP1*^TQ^ seedlings. Values are means ± s.d. (D) Comparison of leaf sheath length (n = 48) together with the relative expression levels of *GNP1* in treated seedling leaf sheaths (n = 6, each with 8 plants) among different uniconazole-P treatments for NIL-*GNP1*^LT^ and NIL-*GNP1*^TQ^ seedlings. Values are means ± s.d.

These results indicate that the accumulation of *GNP1*^LT^ or *GNP1*^TQ^ transcripts (or both) in the plant has a positive effect on grain number and plant height. To determine whether the differences between the *GNP1*^LT^ and *GNP1*^TQ^ promoter regions (S4 Fig) influence *GNP1* expression, and account for the differences in grain number, we analyzed the expression patterns of *GNP1* between NIL-*GNP1*^LT^ and NIL-*GNP1*^TQ^ in different tissues during panicle initiation to the booting stage. *GNP1* was mainly expressed in developing panicles and nodes (S7 Fig), which is consistent with effects of this gene on grain number and plant height. In addition, compared to NIL-*GNP1*^LT^, *GNP1* transcripts were much more abundant in NIL-*GNP1*^TQ^ tissues (S7 Fig). Meanwhile, *GNP1* expression in seedling leaf sheaths was negatively correlated with the dose of GA_3_ used for treatment (Fig 4A and Fig 4C) and positively correlated with that of the GA biosynthesis inhibitor uniconazole-P (Fig 4B and Fig 4D), suggesting that *GNP1* expression is controlled by biologically active GA levels. The *GNP1*^LT^ allele was much more sensitive to uniconazole-P treatment and endogenous GA signal feedback regulation (Fig 4B and Fig 4D), probably due to the sequence variations among promoters. We also investigated *GNP1* expression in the shoot apical meristems and inflorescence meristems. Similar to *OSH1*, a key factor in rice meristem maintenance and regulation, *GNP1* was also expressed in the apical regions of these meristems (S8 Fig). *OSH1* expression signal in NIL-*GNP1*^TQ^ meristems is still strong and specific (S8 Fig), These results suggest that during NIL-*GNP1*^TQ^ inflorescence meristem development, the sequence variations of the promoter might lead to a failure to maintain low *GNP1* expression level, resulting in induced *GNP1* expression in the panicle meristems of NIL-*GNP1*^TQ^.

The above findings demonstrate that the variations in promoters leading to changes in *GNP1* expression in the panicle meristems are the main contributor to the differences in grain number between NIL-*GNP1*^TQ^ and NIL-*GNP1*^LT^. Moreover, the total GNP was positively correlated with the expression level of *GNP1*.

### *GNP1* Influences GA Metabolism

**Fig 5:**
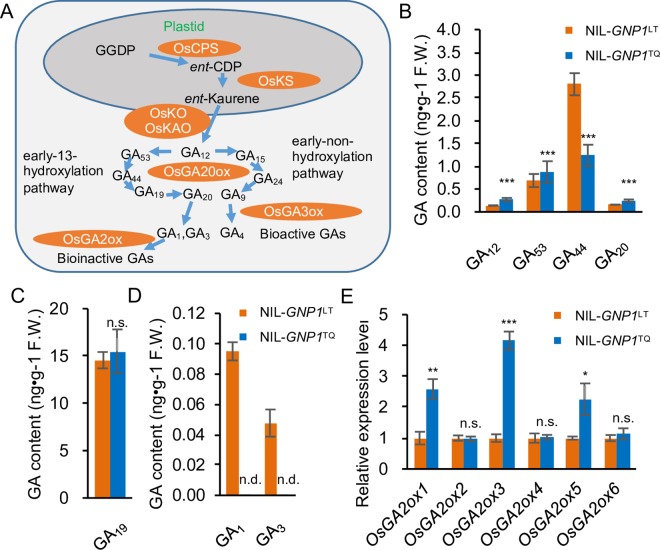
The differences between *GNP1*^TQ^ and *GNP1*^LT^ alleles affect GA biosynthesis. (A) General overview of GA metabolism pathway in higher plants according to previous reports. (B–D) Comparison of the contents of five GA biosynthesis intermediates in the early-13-hydroxylation pathway and two bioactive GAs between young NIL-*GNP1*^LT^ and NIL-*GNP1*^TQ^ panicles (~1 cm) at early panicle initiation to booting stage. Values are means ± s.d. (n = 4, each with 6 plants); n.d., not detected (levels are far too low that beyond the accuracy of detection method). Orange columns, NIL-*GNP1*^LT^; blue columns, NIL-*GNP1*^TQ^. (E) Relative expression levels of rice GA catabolism-related genes in young NIL-*GNP1*^LT^ and NIL-*GNP1*^TQ^ panicles (~1 cm) at early panicle initiation to booting stage. Values are means ± s.d. (n = 4, each with 6 plants). Asterisks represent significant difference determined by Student’s t-test at *p*-value < 0.001 (***), *p*-value < 0.01 (**), *p*-value < 0.05 (*), not significant (n.s.).

*In vitro*, GNP1 (GA20ox1) directly catalyzes the biosynthesis of GA_53_, GA_44_, GA_19_ and GA_20_ in the early-13-hydroxylation pathway with various catalyzing efficiency for each steps [27]. GA_20_ is then used for GA_1_ and GA_3_ biosynthesis via catalyzing by GA3oxs (Fig 5A) [28]. We therefore measured the contents of five endogenous GA biosynthesis intermediates, finding that GA_20_ and GA_12_ accumulated preferentially in the panicle meristems of NIL-*GNP1*^TQ^, whereas GA_44_ levels were much lower and there were no changes in GA_19_ levels relative to NIL-*GNP1*^LT^ (Fig 5B and Fig 5C), indicating that GA_20_ biosynthesis was accelerated. *GNP1* mRNA levels were much higher in NIL-*GNP1*^TQ^, suggesting that the catalytic activity of GNP1 markedly increased as well, leading to higher accumulation of the GA biosynthesis intermediate GA_20_. The increased accumulation of GA_12_ suggests that GA biosynthesis activities including GA_12_ biosynthesis and previous steps might have been activated in this line.

However, in the panicle meristems of NIL-*GNP1*^TQ^, bioactive GA_1_ and GA_3_ were not detected although they were detected in NIL-*GNP1*^LT^ (Fig 5D), indicating that GA_1_ and GA_3_ levels in the NIL-*GNP1*^TQ^ panicle meristems were too low to quantify. Consistent with this result, the GA signal transduction-related genes *RGL3* and *SLR1* were induced in this line (S9 Fig). RGL3 and SLR1 are DELLA proteins and negative regulators of GA signaling, whose degradation by GAs in collaboration with GID1 (gibberellin receptor) [29, 30] and F-box protein is a key event in GA signaling activation [31–33]. Indeed, bioactive GA_1_ and GA_3_ levels were reduced in NIL-*GNP1*^TQ^ panicle meristems. By contrast, most GA biosynthesis-related genes were upregulated, including *OsKAO*, *OsKO*, *OsKS*, *OsCPS* and *OsGA3ox2* (S9 Fig), leading to increased GA_12_ levels (Fig 5B), likely due to feedback activation by reduced bioactive GA (GA_1_ and GA_3_) levels. At the same time, most bioactive GA catabolism genes, i.e., *OsGA2oxs* (Fig 5E), were induced. As GA2oxs directly catalyze progressive catabolic processes that convert active GAs into inactive forms (Fig 5A), the increased catabolic activities in NIL-*GNP1*^TQ^ panicle meristems regulate GA levels much more effectively, regardless of the activated GA biosynthesis process described above. Based on these findings, during NIL-*GNP1*^TQ^ panicle meristem development, GA (GA_1_ and GA_3_) levels happened to be reduced, although the catabolic activities of GNP1 were enhanced.

### GNP1 Activates Cytokinin Activity in the Panicle Meristems

**Fig 6:**
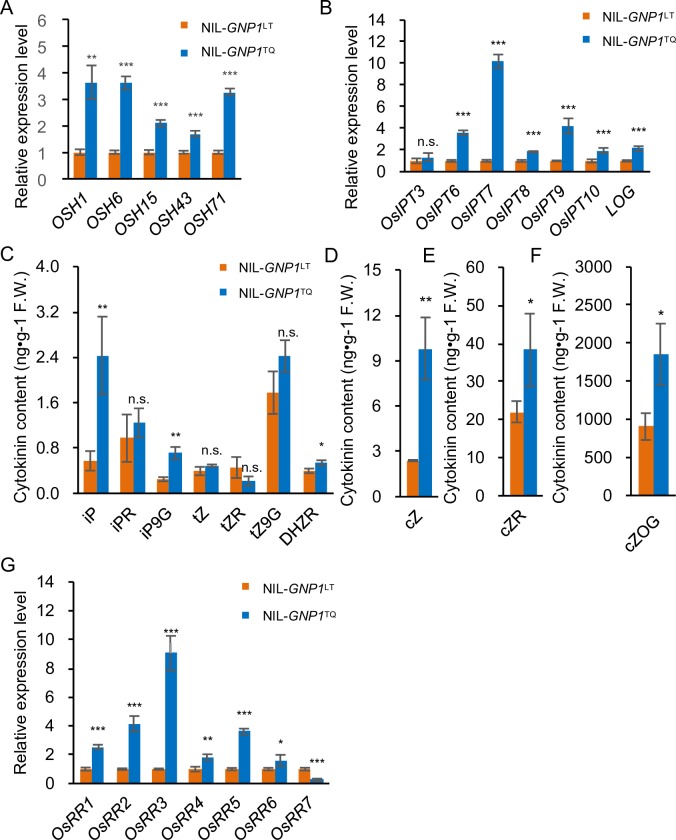
*GNP1*^TQ^ allele activates cytokinin biosynthesis and the cytokinin signal transduction pathway. (A) Relative expression levels of rice *KNOX* genes in young NIL-*GNP1*^LT^ and NIL-*GNP1*^TQ^ panicles (~1 cm) at early panicle initiation to booting stage. Values are means ± s.d. (n = 4, each with 6 plants). (B) Relative expression levels of rice cytokinin biosynthesis-related genes in young NIL-*GNP1*^LT^ and NIL-*GNP1*^TQ^ panicles (~1 cm) at early panicle initiation to booting stage. Values are means ± s.d. (n = 4, each with 6 plants). (C–F) Comparison of endogenous cytokinin levels between young NIL-*GNP1*^LT^ and NIL-*GNP1*^TQ^ panicles (~1 cm) at early panicle initiation to booting stage. Values are means ± s.d. (n = 3, each with 8 plants). Orange columns, NIL-*GNP1*^LT^; blue columns, NIL-*GNP1*^TQ^. (G) Relative expression levels of cytokinin signal transduction-related genes in young NIL-*GNP1*^LT^ and NIL-*GNP1*^TQ^ panicles (~1 cm) at early panicle initiation to booting stage. Values are means ± s.d. (n = 4, each with 6 plants). Asterisks represent significant difference determined by Student’s t-test at *p*-value < 0.001 (***), *p*-value < 0.01 (**), *p*-value < 0.05 (*), not significant (n.s.).

Cytokinins significantly affect reproductive meristem activity [2]. The abnormal GA metabolism in NIL-*GNP1*^TQ^ observed in the current study might be caused by KNOX-mediated responses. To investigate this possibility, we analyzed the expression of five rice *KNOX* genes, including *OSH1*, *OSH6*, *OSH15*, *OSH43* and *OSH71*. The expression of these genes significantly increased in the panicle meristems of NIL-*GNP1*^TQ^ (Fig 6A). *OsIPTs*, which are directly regulated by KNOX proteins, were also upregulated in NIL-*GNP1*^TQ^, as was the cytokinin activating gene *LOG* (Fig 6B), perhaps leading to cytokinin accumulation. We also examined endogenous cytokinins levels in NIL-*GNP1*^TQ^, finding that the levels of several cytokinins and cytokinin biosynthesis intermediates increased in this line (Fig 6C to 6F), leading to increased expression of cytokinin signal response factors (Fig 6G). These results indicate that cytokinin activity was substantially enhanced in NIL-*GNP1*^TQ^ panicle meristems, resulting in increased grain number compared to NIL-*GNP1*^LT^.

## Discussion

### The Role of GNP1 in GA Biosynthesis

A previous *in vitro* study showed that recombinant OsGA20ox1 could catalyze the conversion of GA_12_ and GA_53_ to GA_9_ and GA_20_, but it acts more effectively on GA_53_ [27]. The present study shows that *GNP1* encodes a rice OsGA20ox1 protein. OsGA20ox1 activity is induced via increased expression of *GNP1*, which increases GA_20_ levels *in vivo*. Moreover, *GNP1* transcript levels in seedling leaf sheaths were positively correlated with the treatment dose of uniconazole-P and negatively correlated with that of GA_3_ (Fig 4C and 4D), suggesting that *GNP1* expression is controlled by biologically active GA levels. Moreover, NIL-*GNP1*^LT^ was much more susceptible to endogenous GA signal feedback regulation than NIL-*GNP1*^TQ^, likely due to the sequence variations among promoters leading to altered expression of *GNP1*.

### New Insights into the Regulation of Rice Panicle Meristem Activity by Crosstalk between GAs and Cytokinins

*GNP1* transcripts were mainly detected in newly initiated panicles and in apical regions of meristems overlapping with *OSH1* (a rice *KNOX* gene) expression (S8 Fig). This specific expression pattern implies that GNP1 also plays a fundamental role in regulating panicle meristem activity that is similar to that of cytokinin biosynthesis and signaling genes. The increased grain number of NIL-*GNP1*^TQ^ due to enhanced expression of *GNP1* supports this notion.

**Fig 7:**
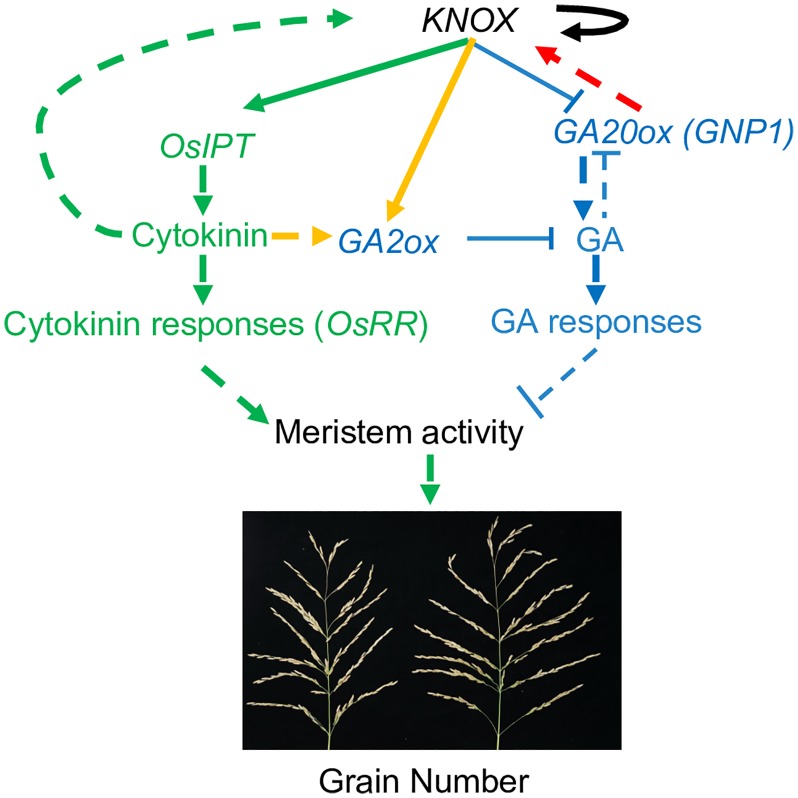
Hypothetical model of the role of the *KNOX* genes-*GNP1* regulatory feedback loop in crosstalk between GA and cytokinin during rice panicle primordium development. Meristem activity and maintenance are regulated via KNOX-mediated GA and cytokinin crosstalk. When NIL-*GNP1*^TQ^ plants transition to the panicle initiation stage, *GNP1* is upregulated in NIL-*GNP1*^TQ^ panicle primordia, which in turn leads to the upregulation of *KNOX* genes (red arrow). The positive autoregulation of *KNOX* genes in the meristem strengthens this regulatory feedback (black arrows). The increased expression of *KNOX* genes activates cytokinin signaling by directly inducing the expression of the cytokinin biosynthesis genes *OsIPTs* (green arrows). Increased cytokinin levels and *KNOX* expression induce the GA catabolism genes *GA2oxs* (orange arrows), in turn leading to enhanced bioactive GA catabolic activity and failed GA_1_ and GA_3_ accumulation in the NIL-*GNP1*^TQ^ inflorescence meristems, thus reducing the detrimental effects of the activated GA biosynthesis pathway (blue arrows) on meristem activity and enhancing meristem activity in NIL-*GNP1*^TQ^ panicle primordia (green arrows). The rebalancing of cytokinin and GA activity in the panicle primordia caused by upregulation of *GNP1* accounts for the increase in grain number per panicle in NIL-*GNP1*^TQ^. In addition, the elevated GA activity in other tissues might account for the increase in plant height. Solid arrows indicate direct regulation, while dashed arrows indicate indirect regulation.

Cytokinins positively regulate reproductive meristem activity [2], GAs are detrimental to meristem activity [20, 21] and KNOX proteins play an irreplaceable role in balancing cytokinin and GA activity in the meristem [25]. We observed increased cytokinin activity in the panicle meristems of NIL-*GNP1*^TQ^, including KNOX-mediated induction of *OsIPTs* and increased levels of cytokinins and cytokinin biosynthesis intermediates, together with enhanced cytokinin responses. In additions, these plants failed to accumulate bioactive GA_1_ and GA_3_ and exhibited significantly increased *KNOX* transcript levels. Taken together, these results demonstrate that increased GNP1 activity positively induces the expression of *KNOX* genes via a feedback loop (Fig 7, red arrow). This promotion of *KNOX* gene expression leads to increased cytokinin activity through directly inducing *OsIPT* expression, as well as upregulation of *GA2oxs*, which negatively regulate GA biosynthesis, thereby reducing GA_1_ and GA_3_ levels. The activation of GA biosynthesis might be due to feedback regulation compensating for the defects in GA_1_ and GA_3_ accumulation, leading to increased accumulation of GA_12_. The tendency for activated GA biosynthesis may be much less effective than that for GA catabolism. This feedback mechanism rebalances cytokinin and GA activity, resulting in increased cytokinin levels and contributing to the higher *GNP1* expression level of NIL-*GNP1*^TQ^.

On the other hand, decreased expression of *GNP1* could lead to lower GA_1_ and GA_3_ level in those positive *GNP1* mimic artificial miRNA transgenic lines, which might eliminate the suppression effect of higher GA_1_ and GA_3_ level on meristem activities, and increase grain number in turn (Fig 7). We propose that during inflorescence meristem development and maintenance processes, increased expression of *GNP1* in those NILs leads to promoted cytokinin activities and gives increased grain number and yield, while decreased expression of *GNP1* in those mimic artificial miRNA transgenic lines most probably contributes to alleviation of the detrimental effect of gibberellins to meristem activity, according to those previous reports, which in turn also gives increased grain number.

### The Use of GNP1 for High Yield Rice Breeding

Numerous efforts aimed at increasing food production to sustain the growing population have focused on elucidating the mechanisms underlying the development of several important agronomic traits in rice, such as panicle architecture. In this study, we cloned a rice *GA20ox1* gene, *GNP1*, whose expression strongly increases rice grain number. Increasing *GNP1* expression may be useful for high yield rice breeding, as these *GNP1* higher-expressed NILs exhibited increased grain number and grain yield, although they were also slightly taller than the controls. When we overexpressed *GNP1* in ZH11, similar results were obtained, thus representing a new strategy for high yield rice breeding.

## Materials and Methods

### Plant Materials

Two sets of reciprocal introgression lines (ILs) derived from a *japonica* rice (*O*. *sativa* L.) variety Lemont and an *indica* variety Teqing were used as materials for QTL mapping [34]. ZH11 and Lemont were used for the transgenic experiments. The *gnp1-D* T-DNA mutant line PFG_2D-41474.R was identified from the Rice Functional Genomic Express Database (RiceGE, http://signal.salk.edu/cgi-bin/RiceGE) and obtained from the Rice T-DNA Insertion Sequence Database (RISD DB, http://cbi.khu.ac.kr/RISD_DB.html) [35]. Oligo sequences used for genotyping the progeny of *gnp1-D* T-DNA insertional line are shown in S4 Table.

### Fine-Mapping of *GNP1*

For map-based cloning of *GNP1*, we performed genotyping of 5,500 BC_5_F_3_ individuals from five BC_5_F_2_ plants that were heterozygous only at the region RM227–RM85, harboring five markers. We identified 16 informative recombinants of four genotypes within this region. Using multiple comparisons of the homozygous recombinant BC_5_F_4_ lines for GNP with the non-recombinant controls, we localized *GNP1* to a 309.5kb region between SL13 and RM85. Further fine mapping using 9,500 BC5F4 plants with six new markers between SL13 and RM85 identified six informative recombinants and four genotypic classes in the target region. We localized *GNP1* to a high-resolution linkage map by progeny testing of BC_5_F_5_ homozygous recombinant plants and narrowed the *GNP1* locus down to a 33.7 kb region between SL65 and SL54. Primers used for fine mapping are shown in S5 Table.

### GA_3_ and Uniconazole-P Treatment

GA_3_ and uniconazole-P treatment were carried out as previously described [36] with minor modifications. For GA_3_ treatment, manually dehulled seeds were sterilized with 75% ethanol for 1 min, washed three times with distilled water, sterilized with 2.5% sodium hypochlorite for 35 min, washed five times with sterile distilled water and incubated on 1/2 MS medium at 4°C for 3 days in the dark. The germinated seeds were transferred to plastic containers containing 1% (w/v) agar with various concentrations of GA_3_ (63492-1G, Sigma-Aldrich).

For uniconazole-P treatment, the seeds were incubated in distilled water with various concentrations of uniconazole-P (19701-25MG, Sigma-Aldrich) at 4°C for 24 h, followed by 26°C for an additional 24 h. The seeds were washed three times with distilled water and incubated for an additional 24 h in distilled water at 26°C. The germinated seeds were grown in 1% (w/v) agar in plastic containers.

Seedlings were grown for 7 days under fluorescent light with a 12 h light/12 h dark photoperiod at 26°C. The second leaf sheath lengths of 48 seedlings per treatment were measured and analyzed. For qRT-PCR analysis, second leaf sheaths were also used, with six pooled replicates for each treatment.

### Plasmid Construction and Plant Transformation

To produce the overexpression constructs, the full-length coding sequence of *GNP1* was amplified from NIL-*GNP1*^TQ^ and cloned into plant binary vector pCAMBIA1300 under the control of single *CaMV 35S* promoter. The artificial microRNA oligo sequences used for *GNP1* silencing were designed as previously described [37] (http://wmd3.weigelworld.org/cgi-bin/webapp.cgi?page=Home;project=stdwmd) and amplified using primer set G-11491 and G-11494. The oligo sequences were inserted into the *XbaI* and *KpnI* sites of pCAMBIA1300 containing one *CaMV 35S* promoter. Oligo sequences for three different target sites were independently used for construction and transformation. The overexpression and silencing plasmids were introduced into *Agrobacterium tumefaciens* strain EHA105 and transferred into the *japonica* variety ZH11.

To produce the construct for the complementary test, 2.2 kb promoter sequence with full-length coding sequences of *GNP1* were amplified from NIL-*GNP1*^LT^. The sequences were then cloned into pCAMBIA1300, introduced into *Agrobacterium tumefaciens* strain EHA105 and used for transformation of NIL-*GNP1*^LT^.

All constructs were confirmed by sequencing. The primer sets are shown in S6 Table, and plant transformation processes were carried out as previously described [38].

### Total RNA Extraction and Real-Time PCR

Total RNA was extracted from various plant tissues using TRIZOL Reagent (Invitrogen). Approximately 500 ng of total RNA was transcribed into first-strand cDNA using ReverTra Ace qPCR RT Master Mix with gDNA Remover (TOYOBO). Real-time PCR data were obtained using an ABI 7300 Real Time PCR System with Fast Start Universal SYBR Green Master Mix with ROX (Roche) and analyzed using the ΔΔCt method. The cycling parameters were 10 min at 95°C, followed by 40 cycles of amplification (95°C for 10 s and 60°C for 1 min). The *ubiquitin* and *actin* genes were used for normalization. The standard amplification slope for real-time PCR primer OsGA2ox1f/OsGA2ox1r was -3.498971, which was used to calculate amplification efficiency. All analyses were repeated at least three times. Primer sets are shown in S7 Table.

### Measuring Endogenous Cytokinin and GA Levels

NIL-*GNP1*^TQ^ and NIL-*GNP1*^LT^ plants were grown in open fields for approximately 5 weeks. Freshly initiated panicles approximately 1 cm long were harvested, and ~1 g samples were used for measurements, with three independent biological repeats per sample. Quantification of endogenous GAs [39] and cytokinins [40] was performed as previously described.

### *In Situ* Hybridization

NIL-*GNP1*^TQ^ plants were grown in open fields for approximately 3 weeks. Samples ~0.5 cm in length including the meristem region were harvested and fixed in 4% (w/v) paraformaldehyde with 0.1% Tween-20, 0.1% Triton-x-100 and 1% (v/v) 25% glutaraldehyde solution in 0.1 M sodium phosphate buffer (pH 7.4) overnight at 4°C. The samples were then dehydrated with a graded ethanol series followed by a dimethylbenzene series. The samples were then embedded in Paraplast Plus (Sigma, P3683), cut into 10 μm sections and mounted on pre-coated poly-prep slides (Sigma, P0425). Digoxigenin-labeled RNA probes were prepared following the instructions of the DIG RNA labeling kit (SP6/T7) (Roche, 11175025910). Hybridization and signal detection were performed as previously described [41]. The primer sets are shown in S8 Table.

### Phenotypic Evaluation of NIL-*GNP1*^TQ^ and Lemont

Yield and related traits for NIL-*GNP1*^TQ^ and the isogenic control (Lemont) were evaluated at five locations: Beijing (40.2°N, 116.2°E); Nanning (22.1°N, 107.5°E), Guangxi province; Jingzhou (30.3°N, 112.2°E), Hubei province; Pingxiang (27.6°N, 113.9°E), Jianxi province and Sanya (18.3°N, 109.3°E), Hainan province, China. NIL-*GNP1*^TQ^ and Lemont plants were grown in a randomized plot design with three replications per line. The area of each plot was 13.2 m^2^, with a single plant transplanted per hill at 25 d after sowing and a spacing of 17 cm between hills and 25 cm between rows. As a basal dressing, 50 kg ha^-1^ each of N, P and K was applied the day before transplanting, and 30 kg ha^-1^ of N was applied twice as topdressing at 1 and 5 weeks after transplanting. At the heading stage, heading date (HD) and plant height (PH) were recorded when 30% of plants contained panicles in each line. At maturity, whole plots were harvested for yield measurements based on a 14% moisture content after air drying. Eight plants were sampled and dried in an oven at 70°C for 5 d for trait investigation, including panicle number per plant (PNP), panicle length (PL), filled grains per panicle (FGP), grain number per panicle (GNP), thousand grain weight (TGW), grain length (GL) and grain width (GW).

### Data Analysis

QTLs affecting GNP were identified using IciMapping 3.0 [42], combined with genotypic data for 157 SSRs and three morphological markers (*Ph*, *gl-1* and *C*) for the ILs [34]. The permutation method was used to obtain empirical thresholds for claiming QTLs based on 1,000 runs in which the trait values were randomly shuffled [43].

## Supporting Information

S1 Fig Frequency distribution in the BC_5_F_3_ population. Frequency distribution of grain number per panicle was derived from a near-isogenic line heterozygous for BC_5_F_2_ at the RM227–RM85 region and confirmed by BC_5_F_4_ family data.
(PDF)

S2 Fig High-resolution mapping of the *GNP1* locus. Black, white and lightly shaded rectangles indicate the homozygous TQ genotype, homozygous LT genotype and marker intervals containing recombination breakpoints, respectively. Different letters (a, b) indicate significant difference determined by the Fisher’s least significant difference (LSD) method at *p*-value < 0.01 (n = 40 plants).
(PDF)

S3 Fig Comparison of agronomic traits. Comparison of (A) plant height, (B) grain length, (C) grain width and (D) 1,000-grain weight and (E) panicle length between NIL-*GNP1*^LT^ and NIL-*GNP1*^TQ^. Values are means ± s.d. (n = 18). Comparison of (F) primary branch number (PBN) and secondary branch number (SBN) between NIL-*GNP1*^LT^ and NIL-*GNP1*^TQ^. Values are means ± s.d. (n = 150). Comparison of (G) primary branch number (PBN) and secondary branch number (SBN) between Lemont (LT) and Teqing (TQ). Values are means ± s.d. (n = 150).
(PDF)

S4 Fig Gene structure and mutation sites of *GNP1* in LT and TQ. Blue bar represents the coding region.
(PDF)

S5 Fig Effect of overexpression of *GNP1* in transgenic rice lines. (A) Gross morphology of three independent *GNP1*^TQ^ overexpression lines and CK (transgenic negative control). Scale bar, 40 cm. (B) Comparison of plant height between three independent *GNP1*^TQ^ overexpression lines and CK. Values are means ± s.d. (n = 10). (C) Gross morphology of two independent *pGNP1*^LT^::*GNP1*^LT^ overexpression lines and the recipient NIL-*GNP1*^LT^. Scale bar, 20 cm. Asterisks represent significant difference determined by Student’s t-test at *p*-value < 0.001 (***).
(PDF)

S6 Fig Effect of knock-down of *GNP1* in transgenic rice lines. (A) Comparison of GNP and FGN between six independent *GNP1* mimic artificial miRNA transgenic lines and CK (transgenic negative control). Values are means ± s.d. (n = 10). (B) Relative expression levels of *GNP1* in the flag leaves of six independent *GNP1* mimic artificial miRNA transgenic lines and CK. Values are means ± s.d. (n = 4). (C) Gross morphology of three independent *GNP1* mimic artificial miRNA transgenic lines and CK. Scale bar, 10 cm. (D) Comparison of plant height between three independent *GNP1* mimic artificial miRNA transgenic lines and CK. Values are means ± s.d. (n = 10). Asterisks represent significant difference determined by Student’s t-test at *p*-value < 0.001 (***), *p*-value < 0.01 (**), *p*-value < 0.05 (*), not significant (n.s.).
(PDF)

S7 Fig Expression patterns of *GNP1* in different tissues at the booting stage. P, premature panicle; N, node; IN, internode; R, root. Values are means ± s.d. (n = 3, each with 4 plants). Asterisks represent significant difference determined by Student’s t-test at *p*-value < 0.001 (***), *p*-value < 0.05 (*).
(PDF)

S8 Fig Expression of *GNP1* in reproductive meristems. (A–C) *In situ* hybridization using an antisense probe for *GNP1* in NIL-*GNP1*^LT^ reproductive meristems at different stages. (A), early stage, with no observable branch meristems at 4 weeks after transplanting; (B), 5 weeks after transplanting, with branch meristems beginning to form. (C), 6 weeks after transplanting, with more branch meristems observed. Scale bar, 100 μm. (D–F) *In situ* hybridization using an antisense probe for *OSH1* in NIL-*GNP1*^LT^ reproductive meristems at different stages. (D), early stage, with no observable branch meristems at 4 weeks after transplanting; (E), 5 weeks after transplanting, with branch meristem beginning to form; (F), 6 weeks after transplanting, with more branch meristems observed. Scale bar, 100 μm. (G) *In situ* hybridization using an antisense probe for *OSH1* in NIL-*GNP1*^TQ^ reproductive meristems at the same stage as that in (E).(H) *In situ* hybridization using a sense probe for *GNP1* at early stages. Scale bar, 100 μm.(I) *In situ* hybridization using a sense probe for *OSH1* at early stages. Scale bar, 100 μm.
(PDF)

S9 Fig Relative expression levels of rice GA biosynthesis-related and signal transduction-related genes in young NIL-*GNP1*^LT^ and NIL-*GNP1*^TQ^ panicles (~1 cm) at early panicle initiation to booting stage. Values are means ± s.d. (n = 4, each with 6 plants). Asterisks represent significant difference determined by Student’s t-test at *p*-value < 0.001 (***), *p*-value < 0.01 (**),not significant (n.s.).
(PDF)

S1 Table QTLs affecting Grain Number per Panicle (GNP) detected in the reciprocal introgression lines (ILs) in Beijing and Sanya. (PDF)

S2 Table The effect of *GNP1* revealed in the BC_5_F_3_ population derived from a near-isogenic line with BC_5_F_2_ heterozygous at the RM227–RM85 region and confirmed by BC_5_F_4_ family data. (PDF)

S3 Table Performance of agronomic traits for NIL-*GNP1*^*TQ*^ and isogenic control (Lemont) across different environments. (PDF)

S4 Table Oligo sequences used for genotyping the progeny of *gnp1-D* T-DNA insertional line. (PDF)

S5 Table Insertion and deletion (Indel) and cleaved amplified polymorphic sequences (CAPS) markers developed. (PDF)

S6 Table Oligo sequences used for transgenic plasmid construction. (PDF)

S7 Table Oligo sequences used for real-time PCR. (PDF)

S8 Table Oligo sequences used for *in situ* hybridization. (PDF)

## References

[1] Virk PS, Khush GS, Peng S. Breeding to enhance yield potential of rice at IRRI: the ideotype approach. Institute Rice Research Notes. 2004;29:S1–S9.

[2] Kyozuka J. Control of shoot and root meristem function by cytokinin. Current opinion in plant biology. 2007;10(5):442–6. 10.1016/j.pbi.2007.08.010 17904411

[3] Ashikari M, Sakakibara H, Lin SY, Yamamoto T, Takashi T, Nishimura A, et al Cytokinin oxidase regulates rice grain production. Science. 2005;309(5735):741–5. 10.1126/science.1113373 15976269

[4] Kurakawa T, Ueda N, Maekawa M, Kobayashi K, Kojima M, Nagato Y, et al Direct control of shoot meristem activity by a cytokinin-activating enzyme. Nature. 2007;445(7128):652–5. 17287810 10.1038/nature05504

[5] Huang X-Y, Chao D-Y, Gao J-P, Zhu M-Z, Shi M, Lin H-X. A previously unknown zinc finger protein, DST, regulates drought and salt tolerance in rice via stomatal aperture control. Gene Dev. 2009;23(15):1805–17. 10.1101/gad.1812409 19651988 PMC2720257

[6] Li S, Zhao B, Yuan D, Duan M, Qian Q, Tang L, et al Rice zinc finger protein DST enhances grain production through controlling Gn1a/OsCKX2 expression. Proc Natl Acad Sci U S A. 2013;110(8):3167–72. 10.1073/pnas.1300359110 23382237 PMC3581943

[7] Ogawa M, Hanada A, Yamauchi Y, Kuwahara A, Kamiya Y, Yamaguchi S. Gibberellin biosynthesis and response during Arabidopsis seed germination. Plant Cell. 2003;15(7):1591–604. 10.1105/tpc.011650 12837949 PMC165403

[8] Chhun T, Aya K, Asano K, Yamamoto E, Morinaka Y, Watanabe M, et al Gibberellin regulates pollen viability and pollen tube growth in rice. Plant Cell. 2007;19(12):3876–88. 10.1105/tpc.107.054759 18083909 PMC2217639

[9] Sakamoto T, Miura K, Itoh H, Tatsumi T, Ueguchi-Tanaka M, Ishiyama K, et al An overview of gibberellin metabolism enzyme genes and their related mutants in rice. Plant Physiol. 2004;134(4):1642–53. 10.1104/pp.103.033696 15075394 PMC419838

[10] Xu J, Shi S, Matsumoto N, Noda M, Kitayama H. Identification of Rgl3 as a potential binding partner for Rap-family small G-proteins and profilin II. Cellular signalling. 2007;19(7):1575–82. 10.1016/j.cellsig.2007.02.004 17382517

[11] Spielmeyer W, Ellis MH, Chandler PM. Semidwarf (sd-1), "green revolution" rice, contains a defective gibberellin 20-oxidase gene. Proc Natl Acad Sci U S A. 2002;99(13):9043–8. 10.1073/pnas.132266399 12077303 PMC124420

[12] Monna L, Kitazawa N, Yoshino R, Suzuki J, Masuda H, Maehara Y, et al Positional cloning of rice semidwarfing gene, sd-1: rice "green revolution gene" encodes a mutant enzyme involved in gibberellin synthesis. DNA research: an international journal for rapid publication of reports on genes and genomes. 2002;9(1):11–7. 10.1093/dnares/9.1.11 11939564

[13] Qin X, Liu JH, Zhao WS, Chen XJ, Guo ZJ, Peng YL. Gibberellin 20-oxidase gene OsGA20ox3 regulates plant stature and disease development in rice. Molecular plant-microbe interactions: MPMI. 2013;26(2):227–39. 10.1094/MPMI-05-12-0138-R 22992000

[14] Itoh H, Ueguchi-Tanaka M, Sentoku N, Kitano H, Matsuoka M, Kobayashi M. Cloning and functional analysis of two gibberellin 3 beta-hydroxylase genes that are differently expressed during the growth of rice. Proc Natl Acad Sci U S A. 2001;98(15):8909–14. 10.1073/pnas.141239398 11438692 PMC37534

[15] Piskurewicz U, Lopez-Molina L. The GA-signaling repressor RGL3 represses testa rupture in response to changes in GA and ABA levels. Plant signaling & behavior. 2009;4(1):63–5. 10.4161/psb.4.1.7331 19704711 PMC2634076

[16] Lo SF, Yang SY, Chen KT, Hsing YI, Zeevaart JA, Chen LJ, et al A novel class of gibberellin 2-oxidases control semidwarfism, tillering, and root development in rice. Plant Cell. 2008;20(10):2603–18. 10.1105/tpc.108.060913 18952778 PMC2590730

[17] Shan C, Mei ZL, Duan JL, Chen HY, Feng HF, Cai WM. OsGA2ox5, a Gibberellin Metabolism Enzyme, Is Involved in Plant Growth, the Root Gravity Response and Salt Stress. Plos One. 2014;9(1). 10.1371/journal.pone.0087110 24475234 PMC3903634

[18] Kaneko M, Itoh H, Inukai Y, Sakamoto T, Ueguchi-Tanaka M, Ashikari M, et al Where do gibberellin biosynthesis and gibberellin signaling occur in rice plants? The Plant journal: for cell and molecular biology. 2003;35(1):104–15. 10.1046/j.1365-313x.2003.01780.x 12834406

[19] Bolduc N, Hake S. The Maize Transcription Factor KNOTTED1 Directly Regulates the Gibberellin Catabolism Gene ga2ox1. Plant Cell. 2009;21(6):1647–58. 10.1105/tpc.109.068221 19567707 PMC2714931

[20] Hay A, Kaur H, Phillips A, Hedden P, Hake S, Tsiantis M. The gibberellin pathway mediates KNOTTED1-type homeobox function in plants with different body plans. Curr Biol. 2002;12(18):1557–65. 10.1016/s0960-9822(02)01125-9 12372247

[21] Sakamoto T, Kamiya N, Ueguchi-Tanaka M, Iwahori S, Matsuoka M. KNOX homeodomain protein directly suppresses the expression of a gibberellin biosynthetic gene in the tobacco shoot apical meristem. Gene Dev. 2001;15(5):581–90. 10.1101/gad.867901 11238378 PMC312643

[22] Tsuda K, Ito Y, Sato Y, Kurata N. Positive Autoregulation of a KNOX Gene Is Essential for Shoot Apical Meristem Maintenance in Rice. Plant Cell. 2011;23(12):4368–81. 10.1105/tpc.111.090050 22207572 PMC3269871

[23] Sakamoto T, Sakakibara H, Kojima M, Yamamoto Y, Nagasaki H, Inukai Y, et al Ectopic expression of KNOTTED1-like homeobox protein induces expression of cytokinin biosynthesis genes in rice. Plant Physiol. 2006;142(1):54–62. 10.1104/pp.106.085811 16861569 PMC1557621

[24] Yanai O, Shani E, Dolezal K, Tarkowski P, Sablowski R, Sandberg G, et al Arabidopsis KNOXI proteins activate cytokinin biosynthesis. Curr Biol. 2005;15(17):1566–71. 10.1016/j.cub.2005.07.060 16139212

[25] Jasinski S, Piazza P, Craft J, Hay A, Woolley L, Rieu I, et al KNOX action in Arabidopsis is mediated by coordinate regulation of cytokinin and gibberellin activities. Curr Biol. 2005;15(17):1560–5. 10.1016/j.cub.2005.07.023 16139211

[26] Zhang D, Yuan Z. Molecular control of grass inflorescence development. Annu Rev Plant Biol. 2014;65:553–78. 10.1146/annurev-arplant-050213-040104 24471834

[27] Toyomasu T, Kawaide H, Sekimoto H, Numers Cv, Phillips AL, Hedden P, et al Cloning and characterization of a cDNA encoding gibberellin 20-oxidase from rice (Oryza sativa) seedlings. Physiologia plantarum. 1997;99(1):111–8. 10.1034/j.1399-3054.1997.990116.x

[28] Yamaguchi S. Gibberellin metabolism and its regulation. Annu Rev Plant Biol. 2008;59:225–51. 10.1146/annurev.arplant.59.032607.092804 18173378

[29] Shimada A, Ueguchi-Tanaka M, Nakatsu T, Nakajima M, Naoe Y, Ohmiya H, et al Structural basis for gibberellin recognition by its receptor GID1. Nature. 2008;456(7221):520–3. 10.1038/nature07546 19037316

[30] Ueguchi-Tanaka M, Ashikari M, Nakajima M, Itoh H, Katoh E, Kobayashi M, et al GIBBERELLIN INSENSITIVE DWARF1 encodes a soluble receptor for gibberellin. Nature. 2005;437(7059):693–8. 10.1038/nature04028 16193045

[31] Ikeda A, Ueguchi-Tanaka M, Sonoda Y, Kitano H, Koshioka M, Futsuhara Y, et al slender rice, a constitutive gibberellin response mutant, is caused by a null mutation of the SLR1 gene, an ortholog of the height-regulating gene GAI/RGA/RHT/D8. Plant Cell. 2001;13(5):999–1010. 10.1105/tpc.13.5.999 11340177 PMC135552

[32] Ueguchi-Tanaka M, Hirano K, Hasegawa Y, Kitano H, Matsuoka M. Release of the repressive activity of rice DELLA protein SLR1 by gibberellin does not require SLR1 degradation in the gid2 mutant. Plant Cell. 2008;20(9):2437–46. 10.1105/tpc.108.061648 18827181 PMC2570727

[33] Ueguchi-Tanaka M, Nakajima M, Katoh E, Ohmiya H, Asano K, Saji S, et al Molecular interactions of a soluble gibberellin receptor, GID1, with a rice DELLA protein, SLR1, and gibberellin. Plant Cell. 2007;19(7):2140–55. 10.1105/tpc.106.043729 17644730 PMC1955699

[34] Mei HW, Xu JL, Li ZK, Yu XQ, Guo LB, Wang YP, et al QTLs influencing panicle size detected in two reciprocal introgressive line (IL) populations in rice (Oryza sativa L.). Theor Appl Genet. 2006;112(4):648–56. 10.1007/s00122-005-0167-0 16331475

[35] Jeong DH, An S, Kang HG, Moon S, Han JJ, Park S, et al T-DNA insertional mutagenesis for activation tagging in rice. Plant Physiol. 2002;130(4):1636–44. 10.1104/pp.014357 12481047 PMC166679

[36] Oikawa T, Koshioka M, Kojima K, Yoshida H, Kawata M. A role of OsGA20ox1, encoding an isoform of gibberellin 20-oxidase, for regulation of plant stature in rice. Plant Mol Biol. 2004;55(5):687–700. 10.1007/s11103-004-1692-y 15604710

[37] Warthmann N, Chen H, Ossowski S, Weigel D, Herve P. Highly specific gene silencing by artificial miRNAs in rice. Plos One. 2008;3(3):e1829 10.1371/journal.pone.0001829 18350165 PMC2262943

[38] Hiei Y, Ohta S, Komari T, Kumashiro T. Efficient transformation of rice (Oryza sativa L.) mediated by Agrobacterium and sequence analysis of the boundaries of the T-DNA. The Plant journal: for cell and molecular biology. 1994;6(2):271–82. 10.1046/j.1365-313x.1994.6020271.x 7920717

[39] Wild M, Achard P. The DELLA protein RGL3 positively contributes to jasmonate/ethylene defense responses. Plant signaling & behavior. 2013;8(4):e23891 10.1046/j.1365-313x.1994.6020271.x 23425858 PMC7030284

[40] Petrovski S, Seviour RJ, Tillett D. Characterization and whole genome sequences of the Rhodococcus bacteriophages RGL3 and RER2. Archives of virology. 2013;158(3):601–9. 10.1007/s00705-012-1530-5 23129131

[41] Langdale J. In situ Hybridization In: Freeling M, Walbot V, editors. The Maize Handbook. Springer Lab Manuals: Springer New York; 1994 p. 165–80.

[42] Li HH, Ye GY, Wang JK. A modified algorithm for the improvement of composite interval mapping. Genetics. 2007;175(1):361–74. 10.1534/genetics.106.066811 17110476 PMC1775001

[43] Churchill GA, Doerge RW. Empirical Threshold Values for Quantitative Trait Mapping. Genetics. 1994;138(3):963–71. 7851788 10.1093/genetics/138.3.963 PMC1206241

