# Supplementary material for: The QTL GNP1 Encodes GA20ox1, Which Increases Grain Number and Yield by Increasing Cytokinin Activity in Rice Panicle Meristems
Source: PLoS Genet. 2016 Oct 20;12(10):e1006386. doi: 10.1371/journal.pgen.1006386 (PMC5072697; doi:10.1371/journal.pgen.1006386)
Supplement: S1 Fig — Frequency distribution of grain number per panicle was derived from a near-isogenic line heterozygous for BC5F2 at the RM227–RM85 region and confirmed by BC5F4 family data. (PDF) [file pgen.1006386.s001.pdf]

S1 Fig

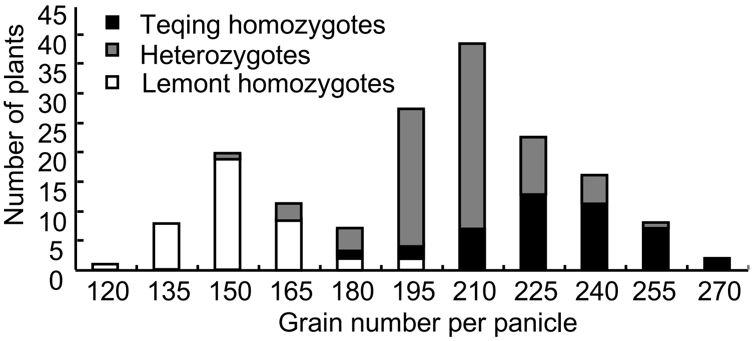

S1 Fig. Frequency distribution in the BC_5F_3 population.

Frequency distribution of grain number per panicle was derived from a near-isogenic line heterozygous for BC_5F_2 at the RM227–RM85 region and confirmed by BC_5F_4 family data.
