# Supplementary material for: The QTL GNP1 Encodes GA20ox1, Which Increases Grain Number and Yield by Increasing Cytokinin Activity in Rice Panicle Meristems
Source: PLoS Genet. 2016 Oct 20;12(10):e1006386. doi: 10.1371/journal.pgen.1006386 (PMC5072697; doi:10.1371/journal.pgen.1006386)
Supplement: S2 Fig — Black, white and lightly shaded rectangles indicate the homozygous TQ genotype, homozygous LT genotype and marker intervals containing recombination breakpoints, respectively. Different letters (a, b) indicate significant difference determined by the Fisher’s least significant difference (LSD) method at p-value < 0.01 (n = 40 plants). (PDF) [file pgen.1006386.s002.pdf]

S2 Fig

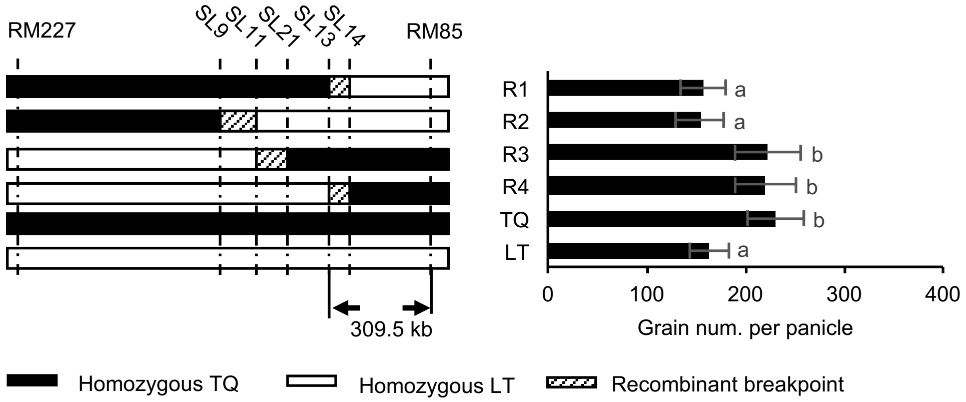

S2 Fig. High-resolution mapping of the *GNP1* locus.

Black, white and lightly shaded rectangles indicate the homozygous TQ genotype, homozygous LT genotype and marker intervals containing recombination breakpoints, respectively. Different letters (a, b) indicate significant difference determined by the Fisher's least significant difference (LSD) method at p-value < 0.01 (n = 40 plants).
