# Supplementary material for: The QTL GNP1 Encodes GA20ox1, Which Increases Grain Number and Yield by Increasing Cytokinin Activity in Rice Panicle Meristems
Source: PLoS Genet. 2016 Oct 20;12(10):e1006386. doi: 10.1371/journal.pgen.1006386 (PMC5072697; doi:10.1371/journal.pgen.1006386)
Supplement: S3 Fig — Comparison of (A) plant height, (B) grain length, (C) grain width and (D) 1,000-grain weight and (E) panicle length between NIL-GNP1LT and NIL-GNP1TQ. Values are means ± s.d. (n = 18). Comparison of (F) primary branch number (PBN) and secondary branch number (SBN) between NIL-GNP1LT and NIL-GNP1TQ. Values are means ± s.d. (n = 150). Comparison of (G) primary branch number (PBN) and secondary branch number (SBN) between Lemont (LT) and Teqing (TQ). Values are means ± s.d. (n = 150). (PDF) [file pgen.1006386.s003.pdf]

S3 Fig

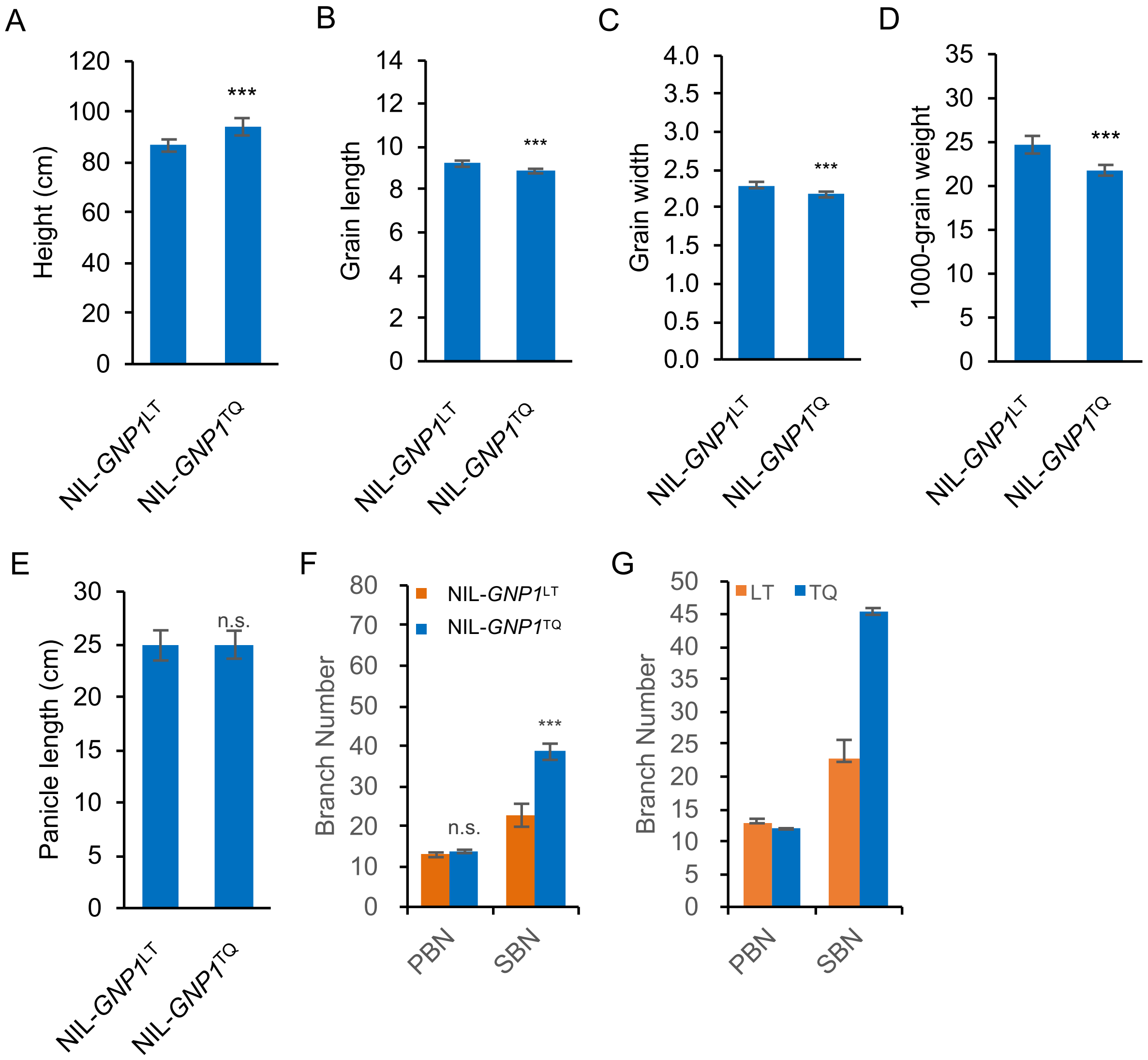

S3 Fig. Comparison of agronomic traits.

Comparison of (A) plant height, (B) grain length, (C) grain width and (D) 1,000-grain weight and (E) panicle length between NIL-*GNP1*^{LT} and NIL-*GNP1*^{TQ}. Values are means ± s.d. (n = 18). Comparison of (F) primary branch number (PBN) and secondary branch number (SBN) between NIL-*GNP1*^{LT} and NIL-*GNP1*^{TQ}. Values are means ± s.d. (n = 150). Comparison of (G) primary branch number (PBN) and secondary branch number (SBN) between Lemont (LT) and Teqing (TQ). Values are means ± s.d. (n = 150).
