# Supplementary material for: The QTL GNP1 Encodes GA20ox1, Which Increases Grain Number and Yield by Increasing Cytokinin Activity in Rice Panicle Meristems
Source: PLoS Genet. 2016 Oct 20;12(10):e1006386. doi: 10.1371/journal.pgen.1006386 (PMC5072697; doi:10.1371/journal.pgen.1006386)
Supplement: S4 Fig — Blue bar represents the coding region. (PDF) [file pgen.1006386.s004.pdf]

S4 Fig

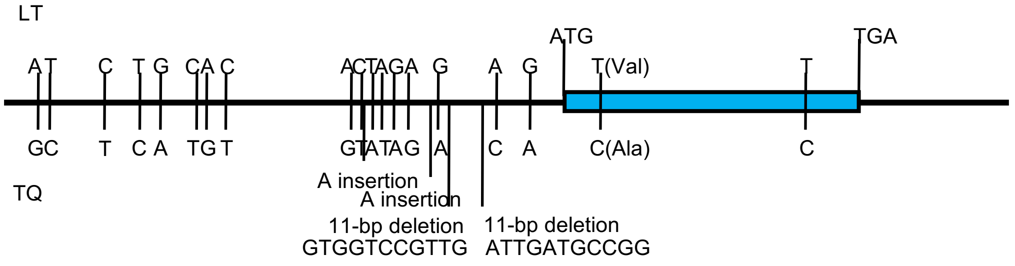

S4 Fig. Gene structure and mutation sites of *GNP1* in LT and TQ.
Blue bar represents the coding region.
