# Supplementary material for: The QTL GNP1 Encodes GA20ox1, Which Increases Grain Number and Yield by Increasing Cytokinin Activity in Rice Panicle Meristems
Source: PLoS Genet. 2016 Oct 20;12(10):e1006386. doi: 10.1371/journal.pgen.1006386 (PMC5072697; doi:10.1371/journal.pgen.1006386)
Supplement: S5 Fig — (A) Gross morphology of three independent GNP1TQ overexpression lines and CK (transgenic negative control). Scale bar, 40 cm. (B) Comparison of plant height between three independent GNP1TQ overexpression lines and CK. Values are means ± s.d. (n = 10). (C) Gross morphology of two independent pGNP1LT::GNP1LT overexpression lines and the recipient NIL-GNP1LT. Scale bar, 20 cm. Asterisks represent significant difference determined by Student’s t-test at p-value < 0.001 (***). (PDF) [file pgen.1006386.s005.pdf]

S5 Fig

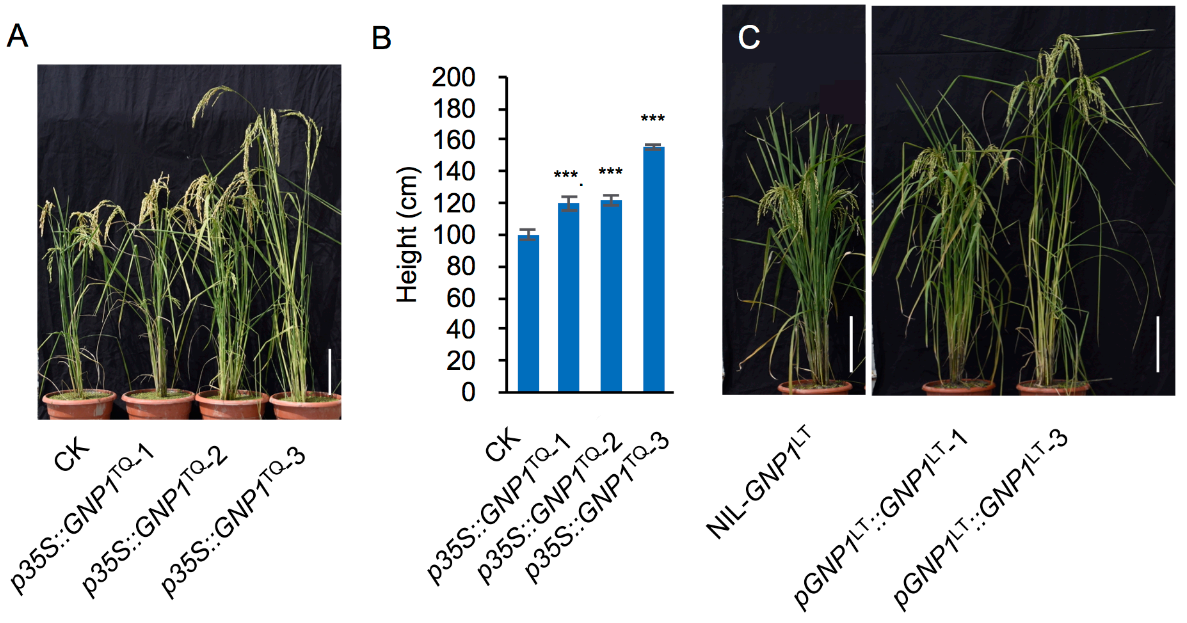

S5 Fig. Effect of overexpression of *GNP1* in transgenic rice lines.
(A) Gross morphology of three independent *GNP1*^{TQ} overexpression lines and CK (transgenic negative control). Scale bar, 40 cm. (B) Comparison of plant height between three independent *GNP1*^{TQ} overexpression lines and CK. Values are means ± s.d. (n = 10). (C) Gross morphology of two independent *pGNP1*^{LT}::*GNP1*^{LT} overexpression lines and the recipient NIL-*GNP1*^{LT}. Scale bar, 20 cm. Asterisks represent significant difference determined by Student's t-test at p-value < 0.001 (***).
