# Supplementary material for: The QTL GNP1 Encodes GA20ox1, Which Increases Grain Number and Yield by Increasing Cytokinin Activity in Rice Panicle Meristems
Source: PLoS Genet. 2016 Oct 20;12(10):e1006386. doi: 10.1371/journal.pgen.1006386 (PMC5072697; doi:10.1371/journal.pgen.1006386)
Supplement: S6 Fig — (A) Comparison of GNP and FGN between six independent GNP1 mimic artificial miRNA transgenic lines and CK (transgenic negative control). Values are means ± s.d. (n = 10). (B) Relative expression levels of GNP1 in the flag leaves of six independent GNP1 mimic artificial miRNA transgenic lines and CK. Values are means ± s.d. (n = 4). (C) Gross morphology of three independent GNP1 mimic artificial miRNA transgenic lines and CK. Scale bar, 10 cm. (D) Comparison of plant height between three independent GNP1 mimic artificial miRNA transgenic lines and CK. Values are means ± s.d. (n = 10). Asterisks represent significant difference determined by Student’s t-test at p-value < 0.001 (***), p-value < 0.01 (**), p-value < 0.05 (*), not significant (n.s.). (PDF) [file pgen.1006386.s006.pdf]

S6 Fig

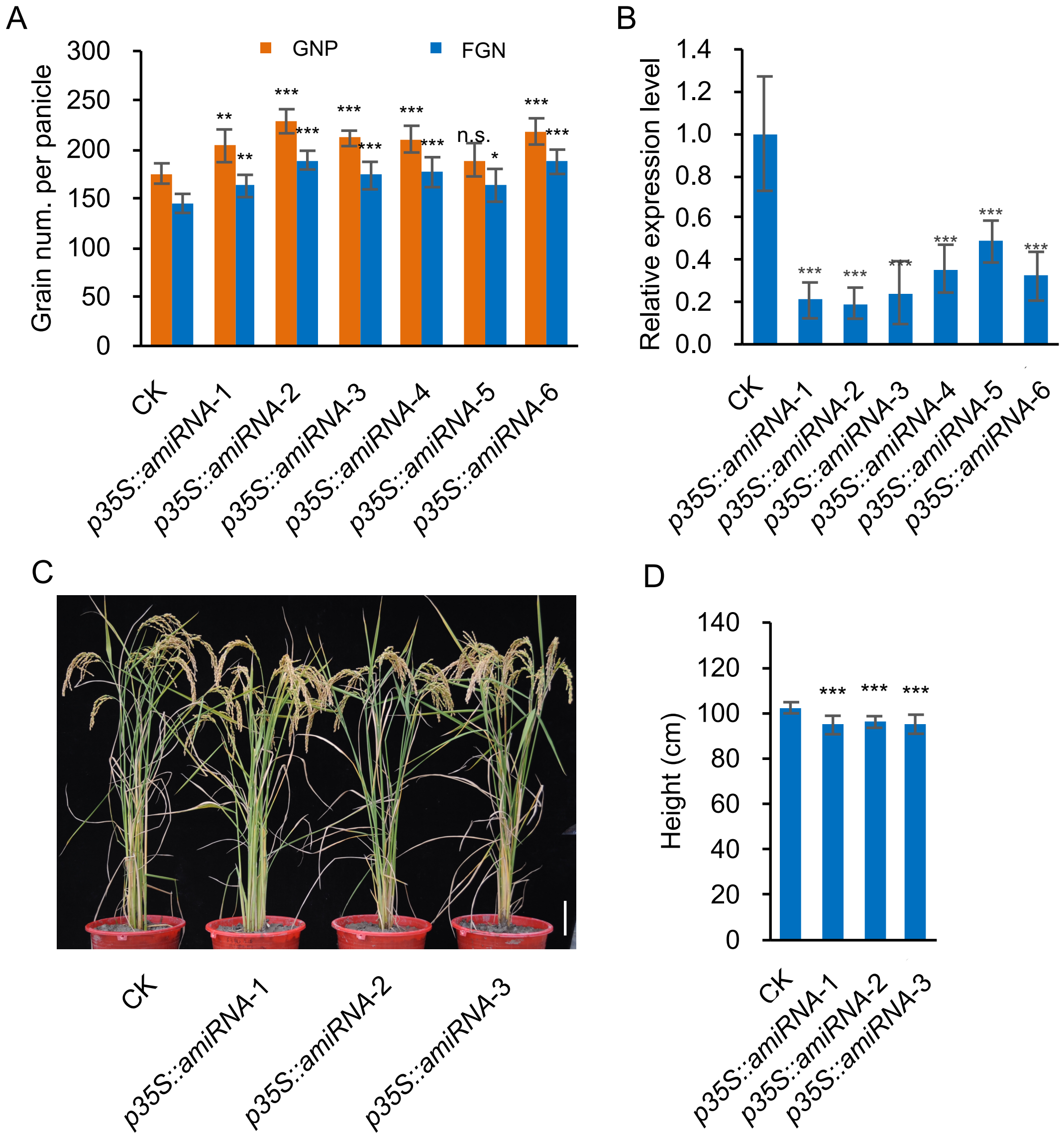

S6 Fig. Effect of knock-down of *GNP1* in transgenic rice lines.

(A) Comparison of GNP and FGN between six independent *GNP1* mimic artificial miRNA transgenic lines and CK (transgenic negative control). Values are means ± s.d. (n = 10). (B) Relative expression levels of *GNP1* in the flag leaves of six independent *GNP1* mimic artificial miRNA transgenic lines and CK. Values are means ± s.d. (n = 4). (C) Gross morphology of three independent *GNP1* mimic artificial miRNA transgenic lines and CK. Scale bar, 10 cm. (D) Comparison of plant height between three independent *GNP1* mimic artificial miRNA transgenic lines and CK. Values are means ± s.d. (n = 10). Asterisks represent significant difference determined by Student's t-test at *p*-value < 0.001 (***), *p*-value < 0.01 (**), *p*-value < 0.05 (*), not significant (n.s.).
