# Supplementary material for: The QTL GNP1 Encodes GA20ox1, Which Increases Grain Number and Yield by Increasing Cytokinin Activity in Rice Panicle Meristems
Source: PLoS Genet. 2016 Oct 20;12(10):e1006386. doi: 10.1371/journal.pgen.1006386 (PMC5072697; doi:10.1371/journal.pgen.1006386)
Supplement: S7 Fig — P, premature panicle; N, node; IN, internode; R, root. Values are means ± s.d. (n = 3, each with 4 plants). Asterisks represent significant difference determined by Student’s t-test at p-value < 0.001 (***), p-value < 0.05 (*). (PDF) [file pgen.1006386.s007.pdf]

S7 Fig

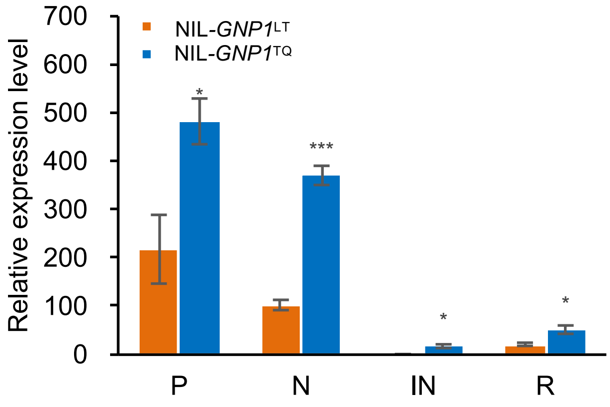

S7 Fig. Expression patterns of *GNP1* in different tissues at the booting stage.

P, premature panicle; N, node; IN, internode; R, root. Values are means ± s.d. (n = 3, each with 4 plants). Asterisks represent significant difference determined by Student's t-test at *p*-value < 0.001 (***), *p*-value < 0.05 (*).
