# Supplementary material for: The QTL GNP1 Encodes GA20ox1, Which Increases Grain Number and Yield by Increasing Cytokinin Activity in Rice Panicle Meristems
Source: PLoS Genet. 2016 Oct 20;12(10):e1006386. doi: 10.1371/journal.pgen.1006386 (PMC5072697; doi:10.1371/journal.pgen.1006386)
Supplement: S8 Fig — (A–C) In situ hybridization using an antisense probe for GNP1 in NIL-GNP1LT reproductive meristems at different stages. (A), early stage, with no observable branch meristems at 4 weeks after transplanting; (B), 5 weeks after transplanting, with branch meristems beginning to form. (C), 6 weeks after transplanting, with more branch meristems observed. Scale bar, 100 μm. (D–F) In situ hybridization using an antisense probe for OSH1 in NIL-GNP1LT reproductive meristems at different stages. (D), early stage, with no observable branch meristems at 4 weeks after transplanting; (E), 5 weeks after transplanting, with branch meristem beginning to form; (F), 6 weeks after transplanting, with more branch meristems observed. Scale bar, 100 μm. (G) In situ hybridization using an antisense probe for OSH1 in NIL-GNP1TQ reproductive meristems at the same stage as that in (E).(H) In situ hybridization using a sense probe for GNP1 at early stages. Scale bar, 100 μm.(I) In situ hybridization using a sense probe for OSH1 at early stages. Scale bar, 100 μm. (PDF) [file pgen.1006386.s008.pdf]

S8 Fig

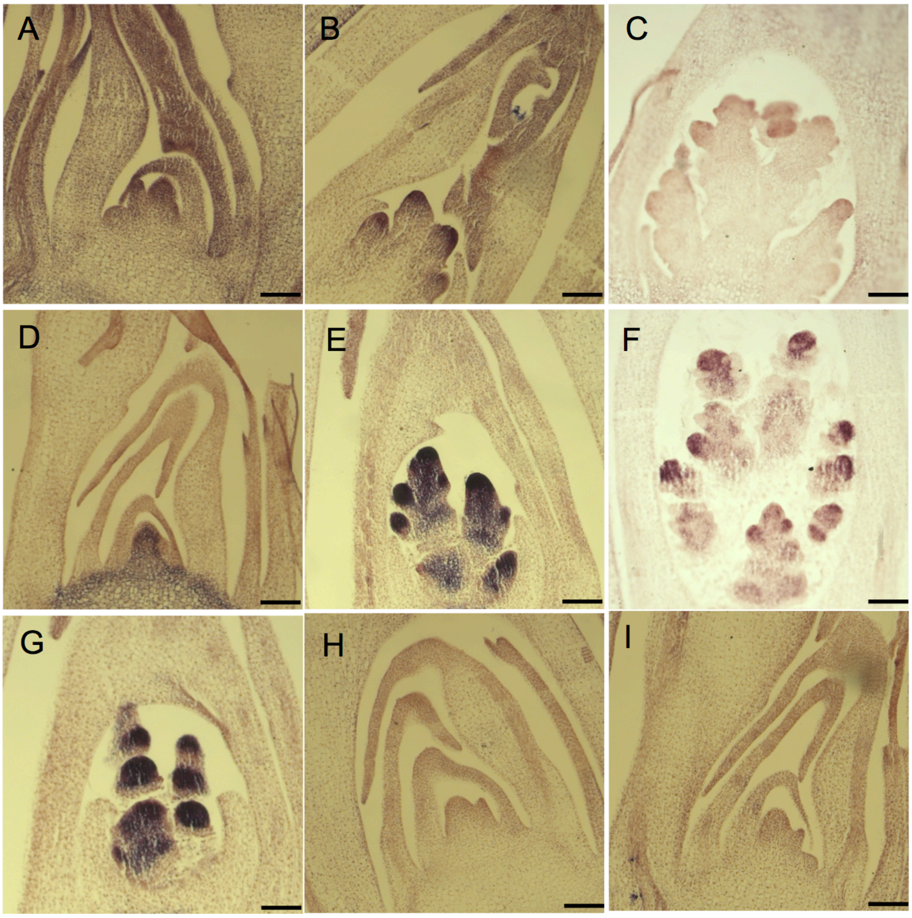

S8 Fig. Expression of *GNP1* in reproductive meristems.

(A–C) In situ hybridization using an antisense probe for *GNP1* in NIL-*GNP1*^{LT} reproductive meristems at different stages. (A), early stage, with no observable branch meristems at 4 weeks after transplanting; (B), 5 weeks after transplanting, with branch meristems beginning to form. (C), 6 weeks after transplanting, with more branch meristems observed. Scale bar, 100 µm. (D–F) In situ hybridization using an antisense probe for *OSH1* in NIL-*GNP1*^{LT} reproductive meristems at different stages. (D), early stage, with no observable branch meristems at 4 weeks after transplanting; (E), 5 weeks after transplanting, with branch meristem beginning to form; (F), 6 weeks after transplanting, with more branch meristems observed. Scale bar, 100 µm. (G) In situ hybridization using an antisense probe for *OSH1* in NIL-*GNP1*^{TQ} reproductive meristems at the same stage as that in (E).(H) In situ hybridization using a sense probe for *GNP1* at early stages. Scale bar, 100 µm. (I) In situ hybridization using a sense probe for *OSH1* at early stages. Scale bar, 100 µm.
