# Supplementary material for: The QTL GNP1 Encodes GA20ox1, Which Increases Grain Number and Yield by Increasing Cytokinin Activity in Rice Panicle Meristems
Source: PLoS Genet. 2016 Oct 20;12(10):e1006386. doi: 10.1371/journal.pgen.1006386 (PMC5072697; doi:10.1371/journal.pgen.1006386)
Supplement: S9 Fig — Values are means ± s.d. (n = 4, each with 6 plants). Asterisks represent significant difference determined by Student’s t-test at p-value < 0.001 (***), p-value < 0.01 (**),not significant (n.s.). (PDF) [file pgen.1006386.s009.pdf]

S9 Fig

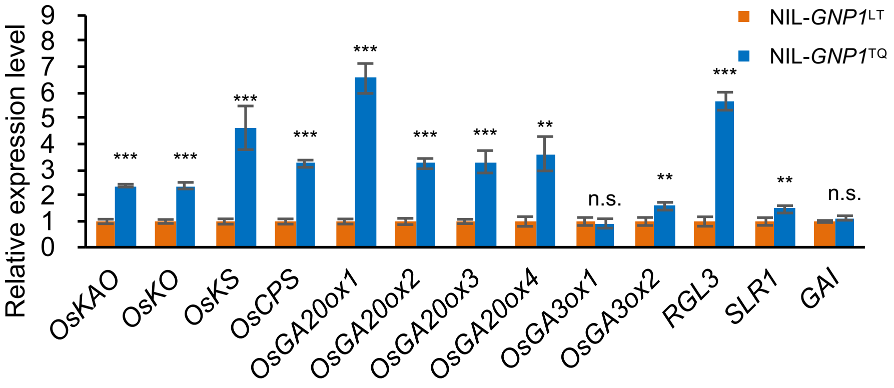

S9 Fig. Relative expression levels of rice GA biosynthesis-related and signal transduction-related genes in young NIL-*GNP1*^{LT} and NIL-*GNP1*^{TQ} panicles (~1 cm) at early panicle initiation to booting stage. Values are means ± s.d. (n = 4, each with 6 plants). Asterisks represent significant difference determined by Student's t-test at *p*-value < 0.001 (***), *p*-value < 0.01 (**),not significant (n.s.).
