# Supplementary material for: The QTL GNP1 Encodes GA20ox1, Which Increases Grain Number and Yield by Increasing Cytokinin Activity in Rice Panicle Meristems
Source: PLoS Genet. 2016 Oct 20;12(10):e1006386. doi: 10.1371/journal.pgen.1006386 (PMC5072697; doi:10.1371/journal.pgen.1006386)
Supplement: S1 Table — (PDF) [file pgen.1006386.s010.pdf]

S1 Table

S1 Table. QTLs affecting Grain Number per Panicle (GNP) detected in the reciprocal introgression lines (ILs) in Beijing and Sanya.

Population	Marker interval[1)]	Environment[2)]	Chr	GNP			
				QTL	LOD	*A*	PVE(%)
TQ-ILs	RM128-OSR23	SY	1	*QGnp1*	4.69	-13.9	5.3
	**RM227–RM85**	**BJ**	**3**	***QGnp3b***	**3.04**	**-19.5**	**4.3**
		**SY**			**6.54**	**-19.8**	**11.3**
	RM255–RM348	BJ	4	*QGnp4b*	6.86	19.3	10.7
		SY			3.39	13.7	4
	RM289–RM163	BJ	5	*QGnp5*	5.14	16.8	8.5
	RM439–RM141	SY	6	*QGnp6b*	2.22	28.8	5.6
	RM210–RM556	BJ	8	*QGnp8b*	4.35	-16.5	6.1
		SY			3.35	-19.4	7.6
	RM147–RM590	SY	10	*QGnp10*	10.35	-22.3	12.9
LT-ILs	RM112–RM208	SY	2	*QGnp2*	5.43	-18.5	9.8
	RM81B–RM231	BJ	3	*QGnp3a*	8.87	-25.3	12.7
	**RM227–RM85**	**BJ**	**3**	***QGnp3b***	**9.59**	**-14.6**	**11.6**
		**SY**			**7.36**	**-21.5**	**12.5**
	RM255–RM348	BJ	4	*QGnp4b*	14.62	21.3	23
		SY			6.1	26.2	12.2
	RM204–RM225	SY	6	*QGnp6a*	4.84	-17.2	7.2
	RM439–RM141	SY	6	*QGnp6b*	5.37	-17.5	8.7
	RM339–RM223	BJ	8	*QGnp8a*	4.97	14.4	6.4
		SY			2.62	14.6	6.5
	RM242–RM278	SY	9	*QGnp9*	4.11	16.4	6.4

1) The underlined markers are those closer to the putative QTL.
2) BJ: Beijing, SY: Sanya
3) The QTL additive effect results from substitution of a Teqing (TQ) allele by a Lemont (LT) allele.
