# Supplementary material for: The QTL GNP1 Encodes GA20ox1, Which Increases Grain Number and Yield by Increasing Cytokinin Activity in Rice Panicle Meristems
Source: PLoS Genet. 2016 Oct 20;12(10):e1006386. doi: 10.1371/journal.pgen.1006386 (PMC5072697; doi:10.1371/journal.pgen.1006386)
Supplement: S2 Table — (PDF) [file pgen.1006386.s011.pdf]

S2 Table

S2 Table. The effect of *GNP1* revealed in the BC_5F_3 population derived from a near-isogenic line with BC_5F_2 heterozygous at the RM227–RM85 region and confirmed by BC_5F_4 family data.

	Genotype	Plant number	X^2(1:2:1)	Phenotype	Additive effect	Dominant effect
GNP	LT homozygotes	41		154.3		
	Heterozygotes	80		203.4		
	TQ homozygotes	42	0.07	229.6	37.7	11.5
