# Supplementary material for: The QTL GNP1 Encodes GA20ox1, Which Increases Grain Number and Yield by Increasing Cytokinin Activity in Rice Panicle Meristems
Source: PLoS Genet. 2016 Oct 20;12(10):e1006386. doi: 10.1371/journal.pgen.1006386 (PMC5072697; doi:10.1371/journal.pgen.1006386)
Supplement: S3 Table — (PDF) [file pgen.1006386.s012.pdf]

S3 Table. Performance of agronomic traits for NIL-*GNP1*^{TQ} and isogenic control (Lemont) across different environments.

Env.	Genotype	PH	PNP	HD
BJ	Lemont	86.9	9.6	98.7
	NIL-*GNP1*^{TQ}	95.1**	8.1**	99.8
NN	Lemont	90.7	4.9	80.7
	NIL-*GNP1*^{TQ}	96.9*	4.9	81.6
JZ	Lemont	97.7	10.7	97.1
	NIL-*GNP1*^{TQ}	110.3***	11.3	95.6
PX	Lemont	93.9	12.6	90.2
	NIL-*GNP1*^{TQ}	102*	11.5	88.7
SY	Lemont	89.8	10.1	88.9
	NIL-*GNP1*^{TQ}	98.4**	8.6**	87.5

PH: plant height (cm), PNP: panicles per plant, HD: heading date (d), BJ: Beijing, NN: Nanning, JZ: Jingzhou, PX: Pingxiang, SY: Sanya.
*, ** and *** represent significance differences at p-value≤ 0.05, 0.01, and 0.001, respectively.
