# Supplementary material for: The QTL GNP1 Encodes GA20ox1, Which Increases Grain Number and Yield by Increasing Cytokinin Activity in Rice Panicle Meristems
Source: PLoS Genet. 2016 Oct 20;12(10):e1006386. doi: 10.1371/journal.pgen.1006386 (PMC5072697; doi:10.1371/journal.pgen.1006386)
Supplement: S4 Table — (PDF) [file pgen.1006386.s013.pdf]

S4 Table

S4 Table. Oligo sequences used for genotyping the progeny of *gnp1-D* T-DNA insertional line.

Name	Sequence 5’-3’
LP	TTCGCAAATTTGTGTGAAGC
RP	AAGTACTCATCCCCAGCAGC
RBP	CCACAGTTTTCGCGATCCAGACTG
