# Supplementary material for: The QTL GNP1 Encodes GA20ox1, Which Increases Grain Number and Yield by Increasing Cytokinin Activity in Rice Panicle Meristems
Source: PLoS Genet. 2016 Oct 20;12(10):e1006386. doi: 10.1371/journal.pgen.1006386 (PMC5072697; doi:10.1371/journal.pgen.1006386)
Supplement: S5 Table — (PDF) [file pgen.1006386.s014.pdf]

S5 Table

**S5 Table. Insertion and deletion (Indel) and cleaved amplified polymorphic sequences (CAPS) markers developed.**

| Marker | Marker type | Forward primer(5'-3') | Reverse primer(5'-3') | Restriction enzyme |
|---|---|---|---|---|
| SL9 | Indel | GAAAATCCACTTGCTCCC | CATCCTTCCATACCTGTTAGA | |
| SL11 | Indel | CAACATACACTCAAATTCCCTG | ACGTCAGCACAACACCCA | |
| SL21 | Indel | CCCAACTAAACCAGACCTT | CTCCAAAACCTATCCTATGC | |
| SL13 | Indel | AGTAGCAGAGGACGAAGAAT | GTCGTGTTTTGAGTCGGT | |
| SL14 | Indel | CTCATAATCGTTGCTACTCATC | CATGCAGACACGGAAATAC | |
| SL65 | Indel | GCGTATTAAGTGGTGGCA | CACGTCCTGAGTCTTATTGG | |
| SL54 | CAPS | CTCCAAATAGGCCAACAA | ATACATCCCGTGCTTCGT | BsmAI |
| SL42 | Indel | TAGAACCTGATTTCACCCAA | TCTTTCTTGCAGCCGAAT | |
| SL40 | Indel | TGAGGTGGTGCCCTAAAC | AGGAAGCCCTGTGATTGG | |
