# Supplementary material for: The QTL GNP1 Encodes GA20ox1, Which Increases Grain Number and Yield by Increasing Cytokinin Activity in Rice Panicle Meristems
Source: PLoS Genet. 2016 Oct 20;12(10):e1006386. doi: 10.1371/journal.pgen.1006386 (PMC5072697; doi:10.1371/journal.pgen.1006386)
Supplement: S6 Table — (PDF) [file pgen.1006386.s015.pdf]

S6 Table

S6 Table. Oligo sequences used for transgenic plasmid construction.

Name	Sequence 5'-3'	Restriction Enzyme Set PCR Product vs (Plasmid)
1. overexpression		
GNP1OEf	ATGAGCATGGTGGTGCAGCAG	(SmaI)
GNP1OEf	ACGCGCCATGGTACTAGGAGTATATTGTTGGTTGCAGGTGAC	NcoI(NcoI)
2. amiRNAi		
GNP1-miR-s	agTATATATCTAAGCCGGAGCTAcaggagattcagtttga	
GNP1-miR-a	tgTAGCTCCGGCTTAGATATATActgctgctgctacagcc	
GNP1-miR*s	ctTAGCTGCGGGTTAGATATATAttcctgctgctaggctg	
GNP1-miR*a	aaTATATATCTAACCCGCAGCTAagagaggcaaaagtgaa	
primer G-11491	TCGGTACCCAGCAGCAGCCACAGCAAAA	KpnI(KpnI)
primer G-11494	TCTCTAGAGCTGCTGATGCTGATGCCAT	XbaI(XbaI)
3. complementary test		
GNP1pf	ACGCGGAGCTCGCTGTTGCGATGGTTGAGACGA	SacI(SacI)
GNP1pr	CCATATGAACTGCGACGGGATCTC	
GNP1cf	GAGATCCCGTCGCAGTTCATATGG	
GNP1cr	ACGCGACGCGTATCGCACCGTGTTTCCGTCC	MluI(MluI)
