# Supplementary material for: The QTL GNP1 Encodes GA20ox1, Which Increases Grain Number and Yield by Increasing Cytokinin Activity in Rice Panicle Meristems
Source: PLoS Genet. 2016 Oct 20;12(10):e1006386. doi: 10.1371/journal.pgen.1006386 (PMC5072697; doi:10.1371/journal.pgen.1006386)
Supplement: S7 Table — (PDF) [file pgen.1006386.s016.pdf]

S7 Table

S7 Table. Oligo sequences used for real-time PCR.

Name	Sequence 5'-3'	Locus ID
ACTINf	TGCTATGTACGTCGCCATCCAG	LOC_Os03g50885
ACTINr	AATGAGTAACCACGCTCCGTCA	
UBIQUITINf	GACGGACGCACCCTGGCTGACTAC	LOC_Os03g13170
UBIQUITINr	TGCTGCCAATTACCATATACCACGAC	
OSH1f	GCTACCTGAGATTGATGCACA	LOC_Os03g51690
OSH1r	CCACCTTCTGACTCTCCGA	
OSH6f	TCCTCCGACGAAGACCAG	LOC_Os01g19694
OSH6r	TTATCTTCTTCCGTGGGATATGGC	
OSH15f	CAAGATCATGGCGCACCC	LOC_Os07g03770
OSH15r	GTACGCCTCCATGAACTGGTC	
OSH43f	ATCAGCTCGATCAGTTCATGG	LOC_Os03g56110
OSH43r	TGCTTCAGCTGCTTGTCAC	
OSH71f	CTGGTGGAACACACATTACCGTTG	LOC_Os05g03884
OSH71r	GAACCGCATGTCCTCCGAT	
OsIPT3f	CCCTACTGACGCACGGAGA	LOC_Os05g24660
OsIPT3r	GCCTCCACCTTCAACTCCA	
OsIPT6f	GTGGGGAAGTGGTAAATGCTGA	LOC_Os07g09220
OsIPT6r	GTGACCACGTCTGGCAAT	
OsIPT7f	GCAGGTGTCCAAGATCCG	LOC_Os05g47840
OsIPT7r	ATGGCGTACAGGTAGTCGTTG	
OsIPT8f	CGGGGAAGACCAAGCTTT	LOC_Os01g49390
OsIPT8r	GAGCGACACCTTGTTCGT	
OsIPT9f	ATGAATCCACAGGTGCCCTA	LOC_Os01g73760
OsIPT9r	GTTACACACTTCATCTAGCAGG	
OsIPT10f	CCAACCAAGCCGATCAACC	LOC_Os06g51350
OsIPT10r	TTCATGCCAGGACTCAAGC	
LOGf	GGCCCAACTAGGAATCCAC	LOC_Os01g40630
LOGr	TGATCGTCCCAAACCAAGC	
OsRR1f	CTTCGCTGGAGTTGCCAT	LOC_Os04g36070
OsRR1r	TCAAGCACACCACAGGTT	
OsRR2f	GACATCGTGCTGACCGACT	LOC_Os02g35180
OsRR2r	ATCGTTCATCTTGAGAGGCTT	
OsRR3f	CGGAGATGACAGGGTTCG	LOC_Os02g58350
OsRR3r	ATTTCATGATGACGCGGTTG	
OsRR4f	CACCAGATAAGCCGCCACA	LOC_Os01g72330
OsRR4r	TGGAGGACAATCTTGGCTT	
OsRR5f	GGGAAGAGGGCATTGGAG	LOC_Os04g44280
OsRR5r	CTTCCTCCAAGCACCTGT	
OsRR6f	CGTCATCGCCAAGATCCTC	LOC_Os04g57720
OsRR6r	CGGGATCTCCTTGAGCTG	
OsRR7f	CTGGAGATATTGGGCTCGGAA	LOC_Os07g26720
OsRR7r	CGCCTTCTTCCAAGCATC	

S7 Table *(continued)*. Oligo sequences used for real-time PCR.

Name	Sequence 5'-3'	Locus ID
OsGA2ox1f	TACGCCAGCAGCTTCACGG	LOC_Os05g06670
OsGA2ox1r	TCCATCAGCTCCAGCGACA	
OsGA2ox2f	TTCAGGGCCGTCAACCAC	LOC_Os01g22920
OsGA2ox2r	ATTTTGTTCCTGAACCGTCGAG	
OsGA2ox3f	TCGTTGCAGGTTCTGACCA	LOC_Os01g55240
OsGA2ox3r	CCTGTTGTCTCCAAGCCTT	
OsGA2ox4f	CTCCTTCCTCGTCATCGTC	LOC_Os05g43880
OsGA2ox4r	GCAGCCTTCTTGTACTCCC	
OsGA2ox5f	CCAGCACCCTCATCGTCA	LOC_Os07g01340
OsGA2ox5r	TGCTTCCTGTACTCGCCGAA	
OsGA2ox6f	GACTTCCTCACCGTCCTG	LOC_Os04g44150
OsGA2ox6r	GCACCTTCTTCCTGTACTCC	
OsKAOf	CCAAGTTTATCCTGACCCCAA	LOC_Os06g02019
OsKAOr	TCGCCAAGCAGTTGTCCA	
OsKOf	GTACCTCAACGCCGTCTTCC	LOC_Os06g37364
OsKOr	CGCCATCGTCTTGTACATGTCC	
OsKSf	CTGCTGCTACCATATTCCCC	LOC_Os04g52230
OsKSr	TTTTGTAACATCACGGCCTT	
OsCPSf	CTACAGGATGCCAATTGTGA	LOC_Os02g17780
OsCPSr	CTCTAGCCCATGCAAGTCG	
OsGA20ox1f	TACGCCAGCAGCTTCACGG	LOC_Os03g63970
OsGA20ox1r	TCCATCAGCTCCAGCGACA	
OsGA20ox2f	GCAACTACTACCCGCCAT	LOC_Os01g66100
OsGA20ox2r	CAGGCAGCTCTTATACCTCC	
OsGA20ox3f	CGCTCACCTTCTTCCTCAACC	LOC_Os07g07420
OsGA20ox3r	AGCCATTCCTTTGCTTGATCCA	
OsGA20ox4f	CGCTTCGTCGACAACCTC	LOC_Os05g34854
OsGA20ox4r	CTGTCCTCGAAGAACTCCC	
OsGA3ox1f	GATCTCTTCCATGTGCTCACC	LOC_Os05g08540
OsGA3ox1r	GAATCATGCTCAACGCCGAT	
OsGA3ox2f	TCCAAGCTCATGTGGTCCGA	LOC_Os01g08220
OsGA3ox2r	TCCTCTCCGCCTCGACTC	
RGL3f	GCCCTTCCAGTTCAACCC	LOC_Os06g10900
RGL3r	GCCACGATGTTCTTGATCTCC	
SLR1f	TGCGGATACTCAACGCCATC	LOC_Os06g03710
SLR1r	CAGCTCCTGCCTCGACTC	
GAIf	GCCTCGTCCACATTGTCG	LOC_Os05g40710
GAIr	CGAAAAACATGCACAGCTTCC	
