# Supplementary material for: The QTL GNP1 Encodes GA20ox1, Which Increases Grain Number and Yield by Increasing Cytokinin Activity in Rice Panicle Meristems
Source: PLoS Genet. 2016 Oct 20;12(10):e1006386. doi: 10.1371/journal.pgen.1006386 (PMC5072697; doi:10.1371/journal.pgen.1006386)
Supplement: S8 Table — (PDF) [file pgen.1006386.s017.pdf]

S8 Table

S8 Table. Oligo sequences used for in situ hybridization.

Name	Sequence 5’-3’
OSH1pSPT18-F	ACGCGAAGCTTGAAGTACAGGGAGGAGCTGACGA
OSH1pSPT18-R	ACGCGTCTAGATAGAGGCCGCCGTCGTTGATG
GNP1pSPT18-F	ACGCGAAGCTTCGGCTACGCCAGCAGCTTCAC
GNP1pSPT18-R	ACGCGTCTAGAGTTGGAGAGCGCCATGAAGGTGTC
